# Transpresentation of interleukin 15 by stromal cell subsets regulates immune cell homeostasis

**DOI:** 10.3389/fimmu.2025.1673309

**Published:** 2026-01-16

**Authors:** Carmen Stecher, Romana Bischl, Elisabeth Potzmann, Anna Schmid-Böse, Stefanie Ferstl, Nina Braun, Ellen R. Richie, Matthias Farlik, Richard A. Flavell, Dietmar Herndler-Brandstetter

**Affiliations:** 1 Center for Cancer Research, Comprehensive Cancer Center Vienna, Medical University of Vienna, Vienna, Austria; 2 Department of Epigenetics and Molecular Carcinogenesis, The University of Texas MD Anderson Cancer Center, Houston TX, United States; 3 Department of Dermatology, Comprehensive Cancer Center Vienna, Medical University of Vienna, Vienna, Austria; 4 Department of Immunobiology, Howard Hughes Medical Institute, Yale University School of Medicine, New Haven CT, United States

**Keywords:** bone marrow niche, CD8 T cells, IL-15Rα, interleukin 15, mesenchymal stromal cells, NK cells

## Abstract

Stromal cells are important bone marrow (BM) niche components that regulate immune cell homeostasis through the production of cytokines such as interleukin 15 (IL-15). Although stromal-derived IL-15 is known to support lymphocyte survival, it remains unclear which stromal cell subsets are capable of IL-15 transpresentation, and how they influence specific lymphocyte populations. By using conditional IL-15 receptor alpha (IL-15Rα) deletion models, we demonstrate that IL-15Rα expression by BM stromal cells is essential for the maintenance of multiple IL-15-dependent lymphocyte populations. Deletion of IL-15Rα in Lepr^+^ or IL-7^+^ stromal cells selectively reduced central memory CD8^+^ T cells in the BM, whereas deletion of IL-15Rα in Osx^+^ stromal cells resulted in a marked loss of natural killer T (NKT) cells and tissue-resident memory CD8^+^ T cells. Surprisingly, endothelial-specific IL-15Rα deletion did not affect lymphocyte maintenance in the BM, but specifically impaired natural killer (NK) maturation and survival in the periphery, uncovering a role of endothelial IL-15 in mature NK cell maintenance. Together, our findings establish that transpresentation of IL-15 by distinct BM stromal cell subsets creates functionally specialized BM niches to support specific lymphocyte populations.

## 1 Introduction

The bone marrow (BM) is a semi-solid tissue within the medullar cavity of the bone. It serves as a reservoir for hematopoietic stem and progenitor cells as well as a diverse range of differentiated immune cell subsets, including long-lived memory T cells, natural killer (NK) cells, invariant NKT cells, and innate lymphoid cells (ILCs) (1, 2). These hematopoietic cells rely on survival signals and homeostatic cues from specialized microenvironments within the BM, which can be provided by stromal cell populations (3). Among the key cytokines that specifically sustain certain lymphocyte populations in the BM is interleukin 15 (IL-15), a pleiotropic cytokine of the common gamma chain (γc) family crucial for the survival and function of memory CD8^+^ T cells, NK cells, and NKT cells (4–6). Unlike other γc cytokines, IL-15 is not commonly secreted under physiological conditions but is instead presented *in trans* by cells expressing IL-15 complexed with its high-affinity receptor alpha chain (IL-15Rα). This unique mode of action, referred to as trans-presentation, requires physical interactions between IL-15-producing cells and responding lymphocytes that express the IL-2/15Rβ and common γc-chain (CD122 and CD132, respectively) (7, 8), resulting in tightly controlled, geographically restricted signaling. This delivery mechanism raises important questions regarding the identity and specialization of IL-15 transpresentation niches within lymphoid and non-lymphoid organs.

The regulation of IL-15 expression itself is complex and operates at multiple levels. At the transcriptional level, IL-15 expression is controlled by various stimuli including type I interferons, TLR ligands, or exposure to IFN-γ, which can differentially induce Il-15 in monocytes, dendritic cells, and stromal cells (9). However, high Il15 mRNA expression does not always correlate with protein production, owing to extensive post-transcriptional and translational control. The 5’ untranslated region (UTR) of IL-15 mRNA contains multiple upstream AUGs that inhibit efficient translation, and the protein is subject to stringent intracellular trafficking and degradation mechanisms (10–13). Similarly, IL-15Rα is regulated not only transcriptionally but also via trans-endosomal recycling, alternative splicing and shedding of its extracellular domain, which can yield soluble IL-15Rα with distinct biological functions (7, 14, 15).

Previous studies have highlighted the importance of IL-15 transpresentation in thymic development (16, 17), NK cell homeostasis (18, 19), and memory T cell survival (5, 20, 21), implicating hematopoietic and non-hematopoietic cells as potential sources of IL-15/IL-15Rα complexes (22). In particular, our recent work demonstrated that mesenchymal stromal cells (MSCs) in the BM produce IL-15 and are required for the maintenance of IL-15-dependent lymphocyte subsets under steady-state conditions (23). However, it is still unclear whether stromal cells merely produce soluble IL-15 or actively trans-present the cytokine via IL-15Rα to adjacent lymphocytes.

In this study, we define the ability of specific BM stromal cell subsets to trans-present IL-15 and to support various lymphocyte compartments *in vivo*. Our results reveal that IL-15Rα expression in Lepr^+^ and IL-7^+^ MSCs is critical for the maintenance of central memory CD8^+^ T cells, whereas Osx^+^ stromal cells are required for the survival of BM-resident NKT cells and tissue-resident memory CD8^+^ T cells. Endothelial cells, while largely dispensable for lymphocyte survival in the BM, were found to influence peripheral NK cell maintenance. By dissecting the stromal architecture of IL-15 delivery, our work provides new insight into the cellular division of labor that sustains lymphocyte diversity in the BM.

## 2 Materials and methods

### 2.1 Experimental animals

Mice were housed in individually ventilated cages (IVCs) under controlled environmental conditions (temperature, humidity, light cycles) with *ad libitum* access to food and water at the Core Facility Laboratory Animal Breeding and Husbandry (Medical University of Vienna). Animal experiments were performed in accordance with institutional and national guidelines and approved by the Austrian Federal Ministry of Education, Science and Research (66.009/0407-V/3b/2018 and 66.009/0408-V/3b/2018).

IL-15-IRES-EGFP knock-in mice were described previously (23). IL-15^flox^ mice were generated by Nan-Shih Liao (Academia Sinica, Taiwan; MTA #13T-1050130-16M) (24) and purchased from the Jackson Laboratory (JAX Stock No. 034188). IL-7 Cre mice (25) were generously provided by Ellen R. Ritchie (MD Anderson Cancer Center). Cdh5-CreERT2 mice (26) were kindly provided by Ralf H. Adams (Max Planck Institute for Molecular Biomedicine, Germany, MTA #151520 with CancerTools.org). CXCL12-DsRed mice (JAX No. 022458), IL-15Rα^flox^ mice (JAX No. 022365), IL-15Rα^KO^ mice (JAX No. 003723), Prx1-Cre mice (JAX No. 005584), Lepr-Cre mice (JAX No. 008320), Osx1-GFP-Cre mice (JAX No. 006361), Rosa26^tm14(tdTomato)^ (JAX No. 007914) and Rosa26^tm1(EYFP)^ mice (JAX No. 006148) were all obtained from the Jackson Laboratory.

To induce the expression of Cre in Cdh5-Cre/ERT2 mice, 1 mg of tamoxifen (Sigma-Aldrich, Cat# T5648) was intraperitoneally injected into mice older than 8-weeks for five consecutive days, and tissues were analyzed 3–6 weeks after the last day of injection.

All Cre-loxP-based conditional knockout experiments were conducted using littermate controls matched for age and sex. Age, number and sex distribution of the mice are indicated in detail in the **Supplementary Table 2**. Cre-driver lines (preferentially males) were backcrossed to floxed mice lacking any Cre-positive ancestry to minimize the risk of unintended recombination. All offspring was systematically screened for germline recombination using deletion-specific genotyping primers (listed in **Supplementary Table 1**).

The Cre driver lines used (Prx1-Cre, Lepr-Cre, Osx-Cre and Cdh5-Cre/ERT2) have been extensively characterized in prior studies (26–30). We previously validated knockout specificity (23) and additionally verified stromal targeting by flow cytometry and immunofluorescence of reporter mice (**Supplementary Figures S6A-E**).

### 2.2 Single cell RNA sequencing and data analysis

For single cell RNA sequencing of CAR cells from mouse long bones, stromal cells were isolated from CXCL12-DsRed^+/-^ mice. BM was flushed from femur and tibia and bones were crushed and digested in Collagenase IV (1mg/mL, Sigma-Aldrich) for 40 minutes to yield a single-cell suspension. Erythrocytes were lysed using ACK buffer (150mM NH_4_Cl, 10mM KHCO_3_, 0.1mM Na_2_EDTA at pH 7.4). Live cells were stained with Aqua Zombie viability dye and sorted on a FACSMelody cell sorter (BD Biosciences) as CD45^-^CD31^-^Ter119^-^DsRed^+^ stromal cells.

Single-cell libraries were then generated using the 10x Genomics Chromium Single Cell 3′ v3 kit, and sequencing was performed across two independent runs. Raw data were processed using CellRanger (version 7.1.0, 10x Genomics).

Data analysis was performed using the Seurat R package (version 5.0.1). Low-quality cells were excluded based on the number of detected features (cells with <200 or >6000 genes were removed) and high mitochondrial gene expression (>10%). Hematopoietic cells were excluded by filtering out cells expressing *Ptprc* and *Gypa*. Datasets were integrated using the Harmony R package, and dimensionality reduction and clustering were performed using UMAP. IL-15 and IL-15Rα-expressing cells were defined as cells with a non-zero expression value for IL-15 or IL-15Rα transcripts, respectively. Clusters with less than 30 cells were excluded from the quantification analysis. Stacked Violin plots were generated using the scCustomize package (31).

Re-analyzed datasets from published bone marrow (32–35) were described previously (23) and derived from Gene Expression Omnibus (GSE156635, GSE122467, GSE108892, GSE128423 and GSE273212).

### 2.3 Tissue preparation

Bone marrow was harvested by flushing femurs and tibiae with FACS buffer (PBS + 2% FBS + 2mM EDTA), followed by red blood cell lysis using ACK buffer (150mM NH4Cl, 10mM KHCO3, 0.1mM Na2EDTA at pH 7.4) and antibody staining for flow cytometric analysis. The spleen was processed by gentle mechanical disruption through a 70 μm cell strainer, followed by ACK lysis and staining. For isolation of immune cells from the liver, left liver lobes where mechanically disrupted through a 70µm cell strainer and then digested in 1mg/mL Collagenase IV and 1mg/mL DNase I (Sigma-Aldrich) in RPMI-1640 medium for 30 minutes at 37 °C. The resulting cell suspension was then topped up with 5mL FACS buffer and centrifuged at 50g for 3 minutes to de-enrich for hepatocytes. The supernatant was pelleted, resuspended in FACS buffer and layered onto Lymphoprep™ medium (Stemcell Technologies) in a Leukosep™ tube (Greiner) to separate mononuclear cells according to the manufacturer’s instructions. Peripheral blood was obtained by terminal retro-orbital bleeding of isoflurane-anaesthesized mice, followed by two rounds of ACK lysis. For endothelial cell analysis, spleens were digested with Collagenase IV (1mg/mL, Sigma-Aldrich) in RPMI-1640 medium for 30 minutes at 37 °C. Cells were then washed, ACK-lysed, and prepared for flow cytometry. To enrich for pericytes, collagenase-digested bone was stained with anti-CD146-PE for 20 minutes and then subjected to magnetic enrichment using the EasySep Mouse PE Positive Selection Kit (StemCell Technologies).

### 2.4 Flow cytometry

Single-cell suspensions were stained with commercial fluorescently conjugated antibodies for 40–60 minutes in 100ul FACS buffer per sample. For *in vivo* labelling of vascular leukocytes, 3µg anti-CD45-PE antibody (Biolegend, 30-F11 Cat#103106) were injected into isoflurane-anaesthesized mice 3–4 minutes before euthanasia and organ isolation (36, 37). The following antibodies were obtained from Biolegend: a4b7 (DATK32, APC, Cat# 120608), CD106 (429, AF647, Cat# 105712), CD117 (2B8, BV785, Cat# 105841); CD117 (ACK2, BV605, Cat# 135122), CD11b (M1/70, PerCP/BV650, Cat# 101230, 101259), CD127 (A7R34, PE, APC, Cat# 135009), CD135 (A2F10, BV421, Cat# 135314), CD144 (BV13, BV421, Cat# 138013), CD146 (ME-9F1, PE, Cat# 134704), CD19 (1D3/CD19, APC-Cy7, Cat# 152412), CD25 (3C7, PE-Cy7, Cat# 101916), CD27 (LG.3A10, FITC, Cat# 124207), CD31 (MEC13.3, PE-Cy7, Cat# 102523), CD3ϵ (145-2C11, BV785, Cat# 100355); CD3ϵ (500A2, APC-Fire750, Cat# 152308), CD3ϵ (17A2, APC, Cat#100236), CD4 (RM4-5, AF700, Cat# 100536), CD44 (IM7, FITC/BV785/BV711, Cat# 103006, 103059, 103057), CD45 (30-F11, AF700/APC-Fire750, Cat# 103128, 103154), CD49a (HM1a, PE-Cy7, Cat# 142608), CD49b (DX5, APC, Cat# 108910), CD54 (YN1/1.7.4, AF647, Cat# 116120), CD62L (MEL-14, PE-Cy7, Cat# 104417), CD69 (H1.2F3, PE/BV605, Cat# 104507), CD71 (R17217, APC-Fire750, Cat# 113828), CD8a (53-6.7, PerCP, Cat# 100732), CXCR3 (CXCR3-173, BV421, Cat# 126522), Eomes (W17001A, PE, Cat# 157705), Gr1 (Rb6-8C5, APC-Cy7, Cat# 108423), KLRG1 (2F1/KLRG1, PE-Cy7, Cat# 138416), NK1.1 (PK136, BV421/APC-Cy7, Cat# 108732, 560618), Sca1 (D7, FITC/BV785, Cat# 108105, 108139), T-bet (4B10, AF488, Cat# 644830), TCRβ (H57-597, APC-Fire750, Cat# 109246), and Ter119 (Ter119, APC-Cy7/FITC/BV421, Cat# 116223, 116206, 116233). CD8a (53-6.7, BB700, Cat# 566410) was obtained from BD Biosciences and LepR (polyclonal biotinylated, Cat# BAF497) from R&D Systems. The Zombie Aqua Viability staining kit (BioLegend, Cat# 423101) was used as a viability dye.

Samples were acquired on a BD LSR Fortessa X-20 or sorted on a FACSMelody cell sorter (BD Biosciences) located at the Center for Cancer Research. Compensation and data analysis was performed using FlowJo (version 10, BD Biosciences). For all samples, quality gates based on size and granularity (FSC-A vs SSC-A gate), doublet exclusion (FSC-A vs FSC-H) and dead cell exclusion (by gating on Zombie Aqua negative cells) were included. The gating strategies for the respective cell types can be found in the **Supplementary Figures**.

### 2.5 ELISA

Levels of IL-15/IL-15Rα complexes were quantified from cell lysates prepared from collagenase-digested bones using a non-denaturing lysis buffer (150 mM NaCl, 50 mM Tris-HCl with 1% Halt Protease Inhibitor Cocktail added before use). Total protein concentration of the lysates was measured using a bicinchoninic assay (BCA) kit (Thermo Fisher Scientific, Cat# 23227). Quantification was performed using the Mouse IL-15/IL-15R Complex Uncoated ELISA kit from Invitrogen (catalog no. 88-7215) according to the manufacturer’s protocol. A five-point standard curve was used for quantification, and data were fitted using five-parameter logistic regression (5PL). Concentrations were adjusted to sample total protein concentration. To account for inter-experimental variation, values were then normalized to the mean of littermate controls within each experiment.

### 2.6 qRT-PCR

RNA was extracted from FACS-sorted cells lysed in RLT Plus lysis buffer supplemented with 1% β-mercaptoethanol using the RNeasy Micro Plus kit from Qiagen. cDNA was synthesized with the RevertAid First Strand cDNA kit from Thermo Fisher Scientific (cat.no. K1621), using oligo-_d_T priming. Quantitative reverse transcription PCR (qRT-PCR) was performed on a CFX96 TouchReal-Time PCR Detection System from BioRad using Maxima SYBR Green/ROX qPCR Master Mix (Thermo Fisher Scientific, cat.no. K0222). Relative expression values were normalized to mouse beta-actin using the comparative threshold cycle method (2−ΔCt). Primers were synthesized by Sigma Aldrich. Primer sequences are listed in **Supplementary Table 1**.

### 2.7 Statistical analysis

Data are represented as mean ± standard error of the mean (SEM) of biological replicates. Statistical analyses were performed using GraphPad Prism version 8. For comparisons between two groups, unpaired two-tailed Student’s *t-tests* were used. A p-value <0.05 was considered statistically significant. Where indicated, p-values between 0.05 and 0.1 are reported above individual graphs. For comparisons involving more than two groups, one-way ANOVA followed by Tukey’s *post hoc* test was applied. Sample processing order was random and group allocation was determined by genotype.

## 3 Results

### 3.1 BM stromal cell subsets differentially express IL-15 and IL-15rα

**Figure 1:**
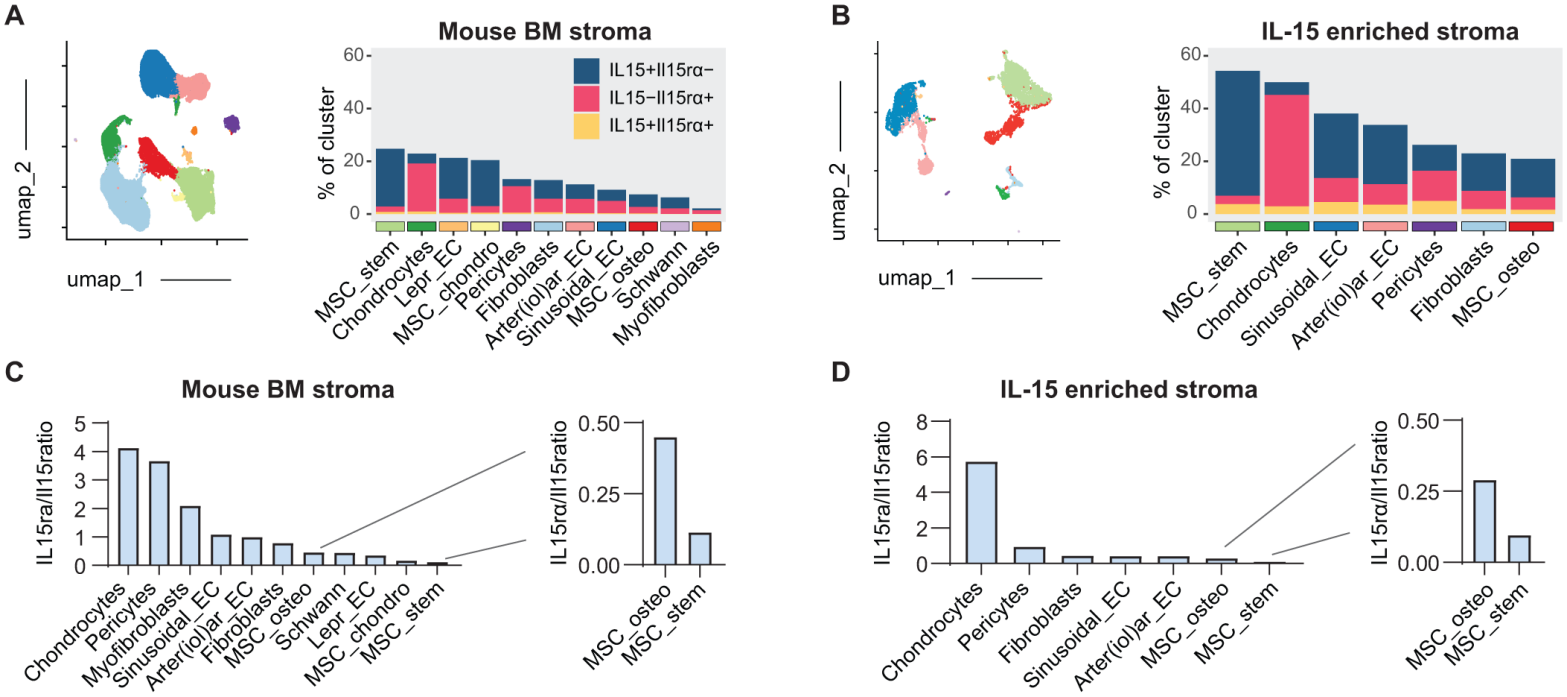
IL-15 and IL-15Rα are differentially expressed by BM stromal cells during the steady state. **(A)** Public scRNA-seq dataset of mouse bone marrow (32–35) showing abundance of cells expressing IL-15, IL-15Rα or both. **(B)** IL-15/IL-15Rα expression of stromal cell clusters in a dataset enriched for IL-15 GFP expressing stroma (23). Clusters containing less than 30 cells were omitted in the quantification plots. **(C)** Fold ratio of IL-15Rα versus IL-15 producers in stromal cell clusters from mouse bone marrow, related to **(A, D)** Fold ratio of IL-15Rα versus IL-15 producers in stromal cell clusters from IL-15 enriched bone marrow, related to B.

We have recently shown that deletion of IL-15 from stromal cells using different conditional knockout mouse models results in a significant reduction of NK cell precursor and memory CD8^+^ T cell populations in the BM (23). It remains unclear whether stromal cells can trans-present IL-15 via IL-15rα, especially since deletion in Lepr^+^ MSCs, which are prominent IL-15 producers, had a small effect on IL-15-dependent immune cell populations in the BM. In fact, when comparing the abundance of IL-15- and IL-15rα-expressing stromal cells in an integrated scRNA-seq dataset from published repositories (32–35) (**Figure 1A**, **Supplementary Figure S1A**) and IL-15-enriched stroma from IL-15^GFP^ mice (23) (**Figure 1B**, **Supplementary Figure S1B**), it becomes apparent that the percentage of cells expressing IL-15, IL-15rα or both, varies greatly across different stromal cell types in the BM. While chondrocytes and pericytes had a very high IL-15rα/IL-15 ratio, mesenchymal stromal cell (MSC) populations, most prominently the CAR-like Lepr^+^ “MSC_stem”, hardly expressed any IL-15rα detectable by scRNA-seq (**Figures 1A, B**). Notably, the ratio between IL-15rα and IL-15 expression was higher in endosteal niche-associated stromal subsets like osteo-MSCs compared to CAR-like MSCs (**Figures 1C, D**). Furthermore, scRNA-seq from sorted Cxcl12^+^ MSCs and endothelial cells using a Cxcl12-DsRed reporter mouse-model indicated that CAR-like MSCs preferentially expressed IL-15 rather than IL-15rα or both IL-15rα and IL-15 (**Supplementary Figures S1C, D**).

Since scRNA-seq data are biased toward underestimating weakly expressed genes (38), the inherently low expression of the IL-15 and IL-15rα transcripts might not be representative of IL-15 complex protein expression *in vivo*. We therefore sorted IL-15^GFP+^ and IL-15^GFP-^ BM macrophages and Lineage^-^CD45^-^CD31^-^ stromal cells from IL-15^GFP^ reporter mice and assessed the expression of IL-15 and IL-15rα via qPCR. IL-15 expression strongly correlated with IL-15rα expression in BM macrophages, but also IL-15^hi^ stromal cells expressed high amounts of IL-15rα mRNA (**Supplementary Figure S1E**).

In summary, there seems to be at least some variation in stromal IL-15rα expression, which raises the question whether Lepr^+^ stromal cells are able to trans-present IL-15 to immune cells, or whether they rather produce soluble IL-15 for IL-15rα-independent signaling.

### 3.2 Lepr^+^ MSCs trans-present IL-15 to central memory CD8^+^ T cells in the BM

**Figure 2:**
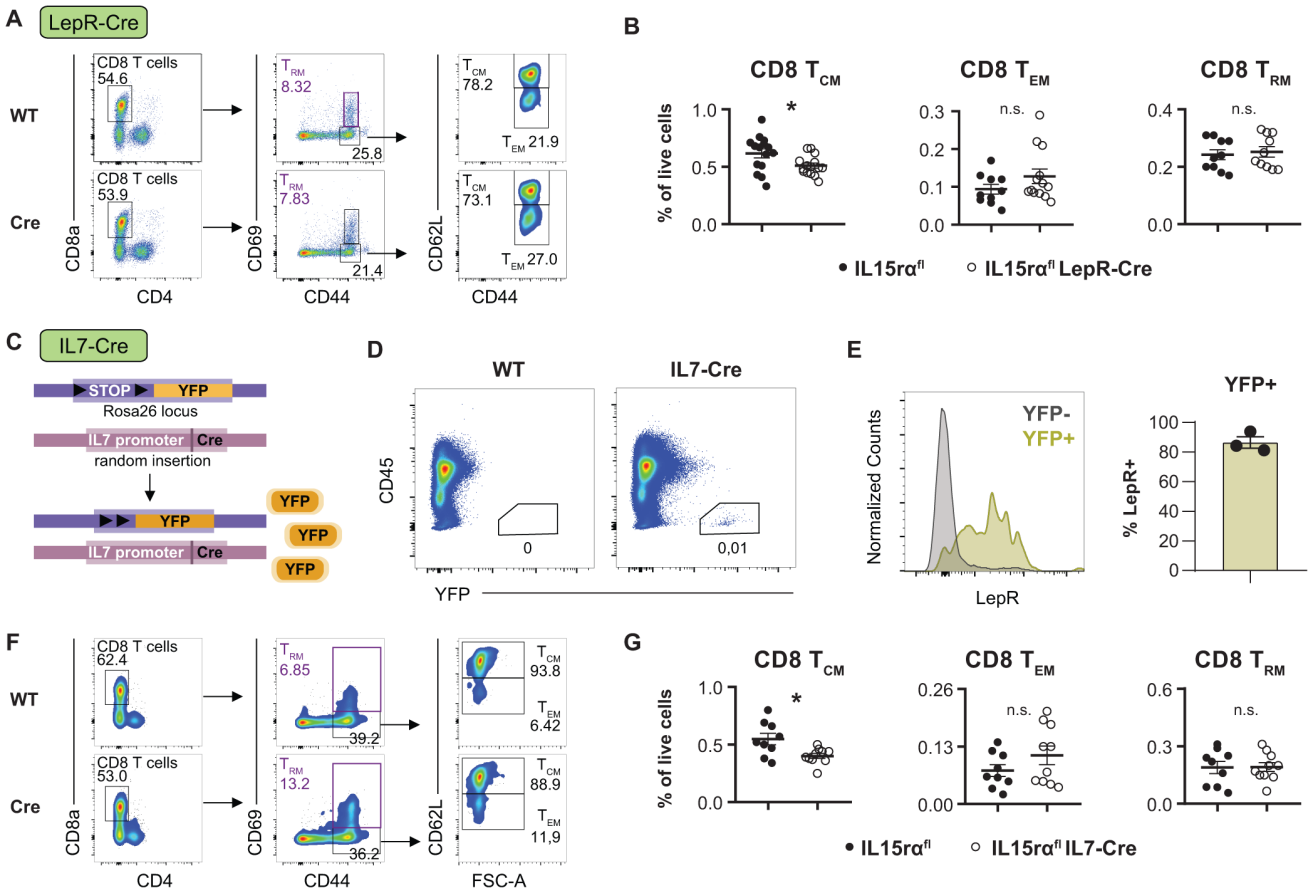
IL-15Rα deletion in IL-7- and Lepr-expressing stromal cells leads to reduced CD8^+^ central memory T cells. Representative flow cytometry plots **(A)** and relative quantification **(B)** of central (T_CM_), effector (T_EM_) and tissue resident (T_RM_) memory CD8^+^ T cells in the bone marrow of IL-15Rα^flox/flox^ Lepr-Cre mice and IL-15Rα^flox/flox^ controls. **(C)** Schematic layout of the generation of an IL-7-Rosa26^YFP^ fate reporter mouse. **(D)** Flow cytometry plots showing YFP^+^CD45^-^ cells in wild type controls and IL-7-Cre Rosa26^YFP^ mice. **(E)** Representative flow cytometry histogram and quantification of LepR expression in YFP^+^ cells. Representative flow cytometry plots **(F)** and relative quantification **(G)** of central (T_CM_), effector (T_EM_) and tissue resident (T_RM_) memory CD8^+^ T cells in the bone marrow of IL-15Rα^flox^ IL-7-Cre mice and IL-15Rα^flox/flox^ littermates. Dot plots show the mean ± SEM with each dot representing a biological replicate. Asterisks above the dot plots indicate *P* values from unpaired two-tailed Student’s *t* tests. *P < 0.05, n.s. *P* > 0.05.

We hypothesized that if stromal cells trans-present IL-15 to immune cells, deletion of IL-15rα should mimic the phenotypes of IL-15 deletion. Thus, we crossed Lepr-Cre mice to IL-15rα^flox^ mice for conditional deletion of IL-15rα from BM MSCs. We have previously established a role of MSC-derived IL-15 in specifically supporting central memory CD8^+^ T cells in the BM, and in supporting an age-dependent NK cell accumulation using Lepr-Cre mice (23). Indeed, central memory but not CD62L^-^ effector memory nor CD69^+^ tissue resident memory CD8^+^ T cells were significantly reduced in the BM of IL-15rα^flox^ Lepr-Cre mice compared to WT littermates (**Figure 2A, B**, **Supplementary Figures S2A, B**). The abundance of NK cells and NKT cells in the BM was unaffected (**Supplementary Figures S2C, D**).

Since central memory T cells depend on both IL-7 and IL-15 for their long term survival (39, 40), we next asked whether IL-7 and IL-15 are provided by the same stromal cells or derived from different stromal niches. In order to track IL-7 expression in the BM, we crossed a Rosa26-YFP strain to IL-7 Cre mice (**Figure 2C**). Within the BM of these IL-7 fate reporter mice, YFP was exclusively expressed in CD45^-^ stromal cells (**Figure 2D**) and the YFP^+^ population strongly overlapped with Lepr^+^ MSCs (**Figure 2E**). On the other hand, IL7^+^ stromal cells represented a distinct subset of Lepr^+^ MSCs (**Supplementary Figure S2E**). Accordingly, scRNA-seq data show a small fraction of IL-7/IL-15 double producers within the stem-like MSC population (**Supplementary Figure S2F**). When crossing IL-15rα^flox^ mice to IL-7-Cre mice, we observed a reduction in central memory but not CD62L^-^ effector memory or CD69^+^ tissue resident CD8^+^ T cells (**Figures 2F, G**), mirroring the Lepr-Cre phenotype and further supporting the observation that CD8 T_CM_, but not T_RM_, rely on MSC-mediated IL-15 transpresentation. IL-15rα^flox^ IL-7-Cre mice further showed a slight reduction of mature Eomes^+^ NK1.1^+^ NK cells, but not NKT cells, in the BM (**Supplementary Figure S2G**). A similar phenotype has previously been observed in aged IL-15^flox^ Lepr-Cre mice (23).

In summary, a polyfunctional MSC population that is capable of producing both IL-7 and IL-15 throughout development appears to specifically support central memory T cells in the BM.

### 3.3 IL-15 trans-presentation by endosteal stromal cells supports tissue resident memory CD8^+^ T cells and NKT cells

Since stromal cells targeted by Osx-Cre have been shown to produce IL-15 important for memory CD8^+^ T cell subsets and NKT cells, we next asked whether Osx^+^ stromal cells were able to transpresent IL-15 via IL-15Rα. We therefore crossed IL-15rα^flox^ with Osx-Cre: GFP mice and assessed immune cell abundances in the BM.

**Figure 3:**
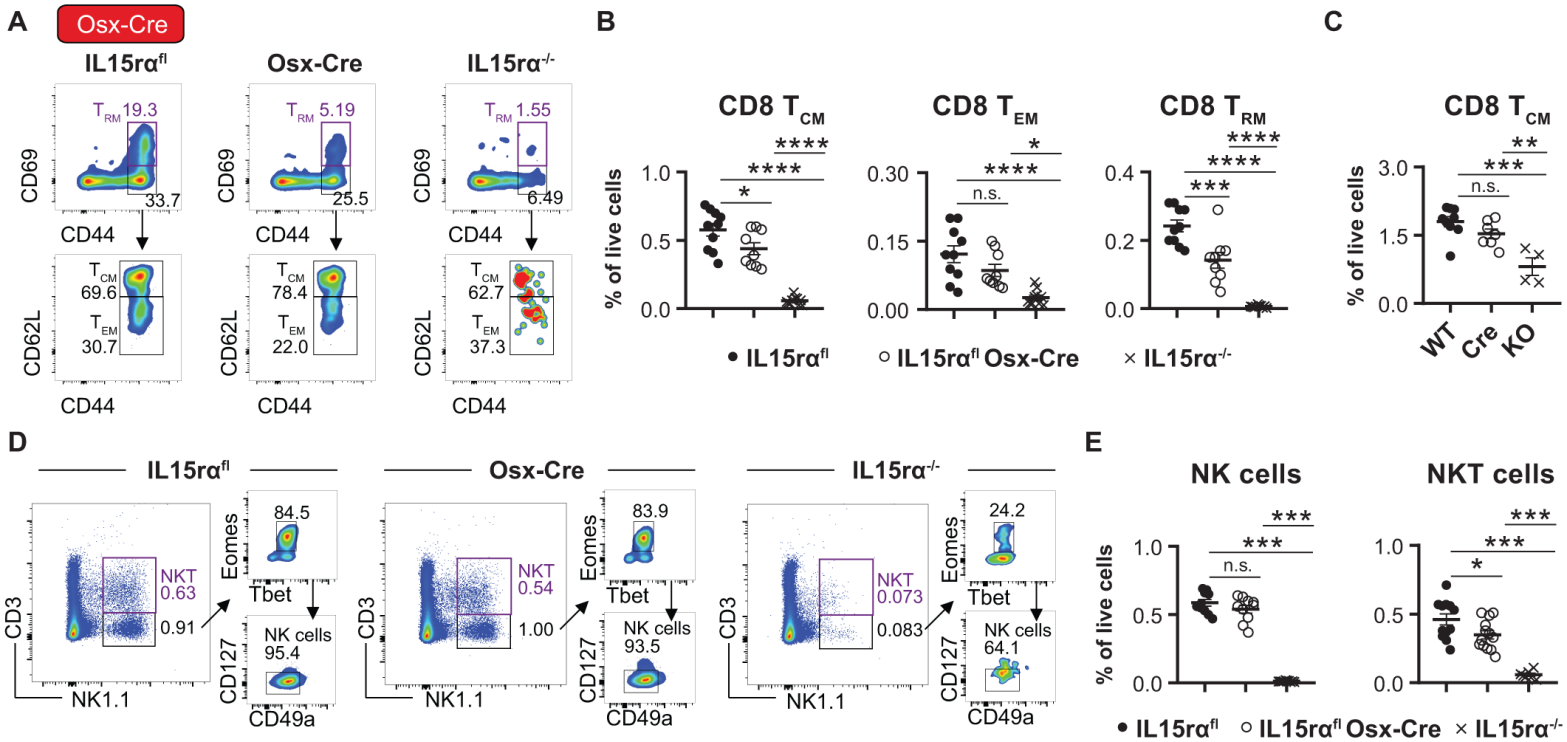
IL-15Rα deletion in Osx-Cre targeted stromal cells leads to loss of memory T cells and NKT cells in the bone marrow. Representative flow cytometry plots **(A)** and relative quantification **(B)** of central (T_CM_), tissue resident (T_RM_), and effector memory CD8^+^ T cells (T_EM_) in the bone marrow of IL-15Rα^flox^ Osx-Cre mice compared to IL-15Rα^flox/flox^ and total knockout (IL-15Rα^KO/KO^) controls. **(C)** Relative quantification of CD44^+^CD62L^+^ central memory CD8^+^ T cells (CD8 T_CM_) in the spleen of IL-15Rα^flox^ Osx-Cre mice compared to IL-15Rα^flox/flox^ littermates. Representative flow cytometry plots **(D)** and relative quantification **(E)** of NK cells and CD3^+^NK1.1^+^ NKT cells in the bone marrow of IL-15Rα^flox^ Osx-Cre mice compared to IL-15Rα^flox/flox^ and total knockout (IL-15Rα^KO/KO^) controls. Dot plots show the mean ± SEM of biological replicates. Asterisks above the dot plots indicate *P* values from Tukey’s multiple comparisons test after One-Way ANOVA. *P < 0.05, **P < 0.01, ***P < 0.001, ****P < 0.0001, n.s. *P* > 0.05.

IL-15rα deletion had a pronounced impact on the abundance of CD8^+^ memory T cells, in particular CD69^+^ tissue resident memory (CD8 T_RM_) cells, although not reaching the levels of IL-15rα^-/-^ mice (**Figures 3A, B**). This phenotype was BM-specific, as neither NK cells nor CD8 T_CM_ cells were reduced in the spleen or blood of IL-15rα^flox^ Osx-Cre mice (**Figure 3C**, **Supplementary Figure S3A–C**). Similarly, the relative abundance of hematopoietic stem cells (LSK) and common lymphoid progenitors (CLP) was not affected (**Supplementary Figures S3D, E**), arguing against a general effect on hematopoiesis. CD3^+^NK1.1^+^ NKT cells, but not CD3^-^NK1.1^+^Eomes^+^CD127^-^ NK cells, were also significantly reduced in the BM of IL-15rα^flox^ Osx-Cre mice compared to WT littermates (**Figures 3D, E**), mirroring the phenotype observed in IL-15^flox^ Osx-Cre mice (23). Since the Osx-Cre transgene is associated with reduced overall bone size and cellularity (**Supplementary Figure S3F**) (41), only relative quantifications have been considered for this strain.

In summary, Osteo-MSCs displayed a higher IL-15rα/IL-15 ratio and Osx-Cre-mediated IL-15rα deletion resulted in a BM-specific reduction of memory CD8^+^ T cells and NKT cells.

### 3.4 IL-15 trans-presentation by Prx1^+^ stromal cells supports NK cell maturation

**Figure 4:**
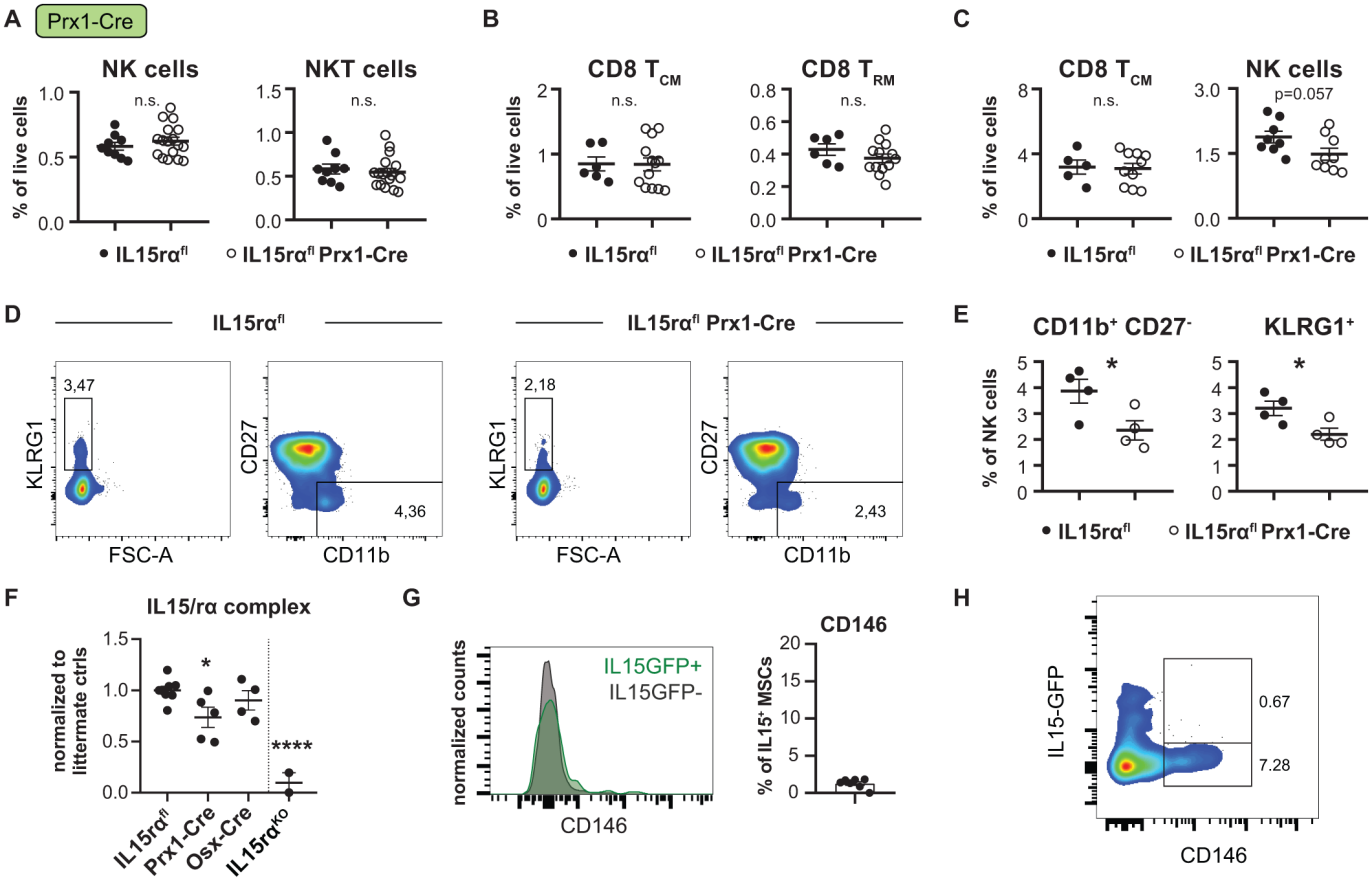
IL-15Rα deletion in Prx1-cre targeted mesenchymal stromal cells affects NK cell maturation. **(A)** Relative quantification of NK cells and NKT cells in the bone marrow of IL-15Rα^flox^ Prx1-Cre mice compared to IL-15Rα^flox/flox^ littermates. **(B)** Relative quantification of central (CD8 T_CM_) and tissue resident memory CD8^+^ T cells (CD8 T_RM_) in the bone marrow of IL-15Rα^flox^ Prx1-Cre mice compared to IL-15Rα^flox/flox^ littermates. **(C)** Quantification of central memory CD8^+^ T cells and NK cells in the spleens of IL-15Rα^flox^ Prx1-Cre mice compared to IL-15Rα^flox/flox^ littermates. Representative flow cytometry plots **(D)** and quantification **(E)** of NK maturation markers. Shown are the relative abundance of the terminally mature CD11b^+^CD27^-^ NK subpopulation among total bone marrow NK cells (pre-gated on CD45^+^Lin^-^SSC-A^low^CD3^-^NK1.1^+^) as well as the percentage of Klrg1^+^ NK cells. **(F)** ELISA comparing IL15/IL15RA complex (quantified as pg/mg total protein and normalized to littermate controls) in the bone marrow of IL-15Rα^flox/flox^ controls, IL-15Rα^flox/flox^ Prx1-Cre mice, IL-15Rα^flox/flox^ Osx-Cre mice and IL-15Rα^KO/KO^ mice. **(G)** Representative flow cytometry histogram (left) and quantification (right) of CD146 expression among IL-15^GFP+^ stromal cells. **(H)** Flow cytometry plot from digested bone of IL-15^GFP^ mice pre-enriched for CD146 (*Mcam*) and pre-gated on CD31^-^Lin^-^CD45^-^ stromal cells showing lack of IL-15-GFP expression in CD146^+^ cells. Dot plots show the mean ± SEM of biological replicates. Asterisks above the dot plots indicate *P* values from unpaired two-tailed Student’s *t* tests. *P < 0.05, ****P < 0.0001, n.s. *P* > 0.05.

Given that Prx1-Cre targets all mesenchymal cells, we anticipated a greater or at least comparable effect to that observed with the previously used Lepr-Cre and Osx-Cre lines. Similar to IL-15 deletion from Prx1-targeted cells (23), NK cells and NKT cells were not affected in the BM of IL-15rα^flox^ Prx1-Cre mice (**Figure 4A**). In contrast to previous observations in IL-15^flox^ Prx1-Cre mice, we surprisingly observed no reduction of memory CD8 T cells in the BM of IL-15rα^flox^ Prx1-Cre mice (**Figure 4B**, **Supplementary Figure S4A**) (23). In the spleen, there was a trend (not reaching statistical significance) toward less NK cells but not memory CD8^+^ T cells (**Figure 4C**, **Supplementary Figures S4B, C**), which fits the phenotype observed with IL-15 deletion (23). Accordingly, no changes in IL-15-dependent lymphocytes were observed in the blood of IL-15rα^flox^ Prx1-Cre mice (**Supplementary Figure S4D**). BM NK cells, although not differing in overall levels, also expressed significantly less KLRG1, and less of the NK cells were of the fully mature CD11b^+^CD27^-^ phenotype (**Figures 4D, E**, **Supplementary Figures S4E, F**). Additionally, BM lysates from IL-15rα^flox^ Prx1-Cre, but not IL-15rα^flox^ Osx-Cre mice, contained significantly less overall IL-15 complexed to IL-15rα (**Figure 4F**).

Because this partial phenocopy was unexpected, we next explored whether certain stromal subsets targeted by Prx1-Cre but not by Osx-Cre or Lepr-Cre might express IL-15Rα in the absence of IL-15, thereby potentially exerting a cytokine scavenging rather than transpresenting role. IL-15rα expression has previously been shown to be able to exert anti-inflammatory, scavenging effects counteracting the effects of IL-15 (42, 43). Based on our transcriptional data (**Figure 1B**), chondrocytes and pericytes showed the highest IL-15rα/IL-15 ratios. While chondrocytes have been shown to produce IL-15 (23) and are also targeted by Osx-Cre (44), pericytes appeared as a candidate stromal cell population expressing IL-15Rα without corresponding IL-15 production. Indeed, pericytes were under-represented among IL-15^GFP+^ sorted stromal cells (23) (**Figure 1B**) and IL-15^GFP+^ Lin^-^ CD45^-^CD31^-^ stromal cells were mostly negative for the pericyte marker *Mcam* (CD146) (**Figure 4G**). Similarly, in CD146- enriched stromal fractions from digested bone of IL-15^GFP^ mice, IL-15^GFP^ expression was absent from the *Mcam*-positive population, suggesting that BM pericytes are IL-15rα^+^ and IL-15^-^, and could thereby locally modulate IL-15 bioavailability (**Figure 4H**).

### 3.5 IL-15 transpresentation regulates endothelial-NK cell interaction and NK cell survival

**Figure 5:**
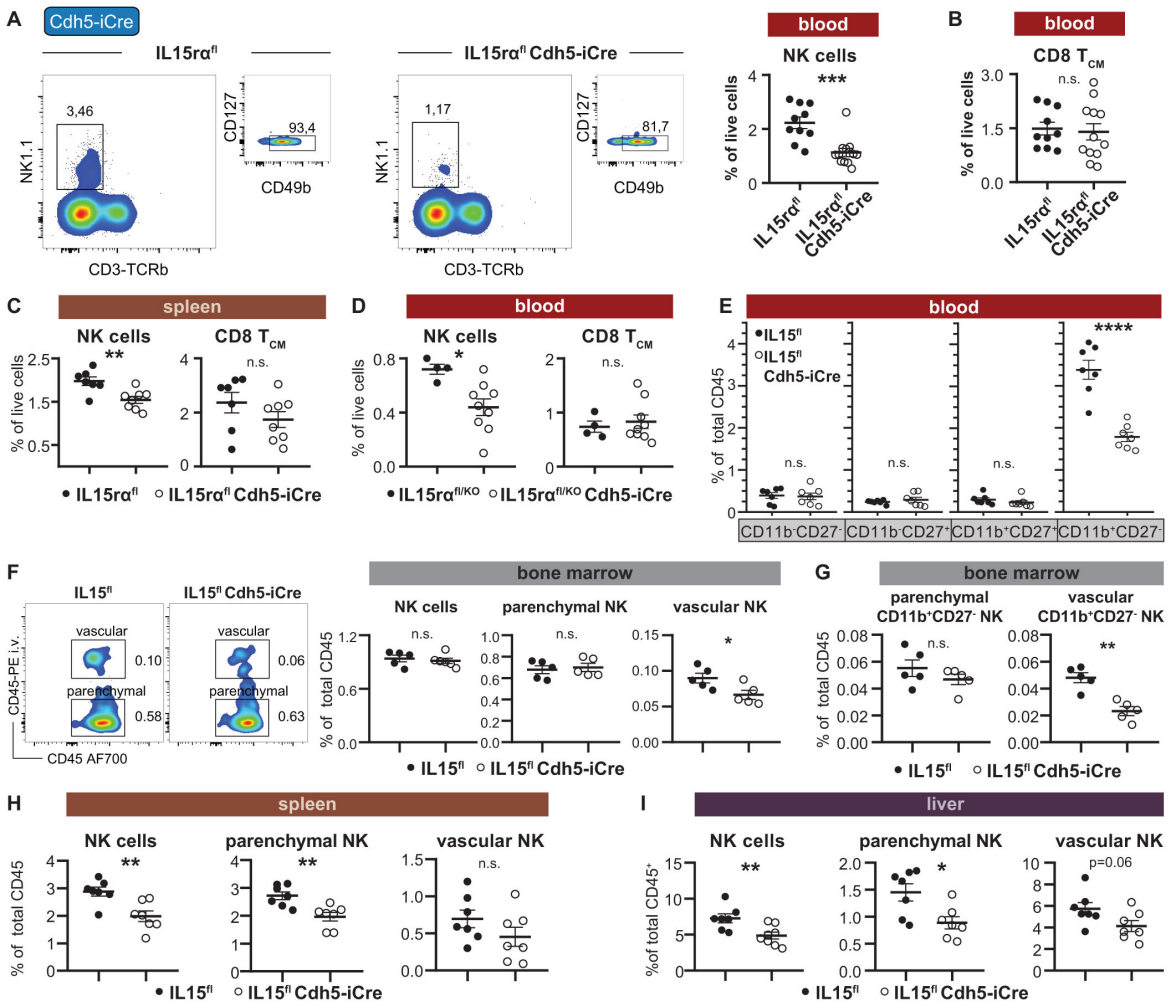
Endothelial IL-15Rα regulates peripheral NK cells and adhesion molecule expression. **(A)** Representative flow cytometry plot and relative quantification of NK cells from the blood of tamoxifen-induced IL-15Rα^flox/flox^ Cdh5-Cre/ERT2 (Cdh5-iCre) mice compared to IL-15Rα^flox/flox^ littermates. All mice were injected with 1mg tamoxifen i.p. for 5 consecutive days, and analyzed 3 to 6 weeks later. **(B)** Relative abundance of central memory CD8^+^ T cells (CD8 T_CM_) in the blood of tamoxifen-induced IL-15Rα^flox/flox^ Cdh5-Cre/ERT2 (Cdh5-iCre) mice compared to IL-15Rα^flox/flox^ littermates (IL-15Rα^fl^). **(C)** Relative quantification of (NK1.1^+^CD3^-^CD49b^+^CD127^-^) NK cells and central memory CD8^+^ T cells in the spleen of IL-15Rα^flox/flox^ Cdh5-Cre/ERT2 (Cdh5-iCre) mice compared to IL-15Rα^flox/flox^ littermates. **(D)** Relative quantification of NK cells and central memory CD8^+^ T cells in the blood of IL-15Rα^flox/KO^ Cdh5-Cre/ERT2 (Cdh5-iCre) mice on a mixed B6/129S background compared to IL-15Rα^flox/KO^ littermates. **(E)** Relative abundance of the indicated NK cell maturation stages in the blood of tamoxifen-induced IL-15^flox/flox^ Cdh5-iCre mice compared to IL-15^flox/flox^ controls. **(F)** Representative flow cytometry plot (left) and quantification (right) of bone marrow NK1.1^+^CD3^-^ NK cells in IL-15^flox/flox^ and IL-15^flox/flox^ Cdh5-iCre mice, subdivided into parenchymal (CD45-PE^-^) and vascular NK cells (CD45-PE^+^). **(G)** Relative abundance of parenchymal (CD45-PE^-^) and vascular (CD45-PE^+^) mature CD11b^+^CD27^-^ NK cells in the bone marrow of IL-15^flox/flox^ Cdh5-iCre mice compared to IL-15^flox/flox^ littermates. **(H)** Relative quantification of splenic NK1.1^+^CD3^-^ NK cells in IL-15^flox/flox^ versus IL-15^flox/flox^ Cdh5-iCre mice, subdivided into parenchymal (CD45-PE^-^) and vascular NK cells (CD45-PE^+^). **(I)** Relative quantification of NK1.1^+^ NK cells int the livers of IL-15^flox/flox^ and IL-15^flox/flox^ Cdh5-iCre mice, subdivided into parenchymal (CD45-PE^-^) and vascular NK cells (CD45-PE^+^). One animal was excluded from the quantification due to loss during the CD45-PE labeling procedure. Dot plots show the mean ± SEM of biological replicates and asterisks above the plots indicate *P* values from unpaired two-tailed Student’s *t* tests. *P < 0.05, **P < 0.01, ***P < 0.001, n.s. *P* > 0.05.

Endothelial cells produce IL-15 in both mice and humans (23, 45, 46). However, deleting mouse IL-15 from endothelial cells has no effect on immune cell abundances in the BM (23), and neither did IL-15rα deletion in the BM when using tamoxifen-inducible Cdh5-iCre mice (**Supplementary Figures S5A, B**). Accordingly, BM lysates from IL-15rα Cdh5-iCre mice had unchanged total amounts of IL-15/IL-15rα complex (**Supplementary Figure S5C**). However, endothelial cell-specific deletion of IL-15rα led to significantly reduced NK cells, but not central memory CD8^+^ T cells, in the blood (**Figures 5A, B**, **Supplementary Figures S5D, E**) and spleen (**Figure 5C**, **Supplementary Figure S5D**). Notably, blood NK cell numbers were further reduced in Cdh5-iCre mice on an IL-15rα^flox/KO^ background compared to their IL-15rα^flox/KO^ littermate controls (**Figure 5D**), suggesting a gene dosage-dependent effect of IL-15rα loss. When comparing circulating NK cells from IL-15^flox^ conditional knockout mice, Cdh5-iCre littermates showed a lower expression of the cell adhesion marker CD44 (**Supplementary Figure S5E**), suggesting a potential role for endothelial IL-15 in NK cell tissue retention and/or trafficking. On the other hand, endothelial cells from the spleen of IL-15^flox^ Cdh5-iCre mice showed a trend towards lower ICAM-1 expression (**Supplementary Figures S5F, G**). To further explore the potential impact of endothelial IL-15 on NK cell trafficking or maturation, we examined expression of the CXC chemokine receptor 3 (CXCR3), which was increased among total NK cells from IL-15^flox^ Cdh5-iCre mice across several compartments, including the blood, spleen and bone marrow (**Supplementary Figure S5H**). However, since Cxcr3 is more prevalent on immature NK cells, this change might represent a compositional shift rather than altered chemokine responsiveness. Consistent with this, the loss of NK cells in the blood specifically affected mature CD11b^+^CD27^-^ NK cells (**Figure 5E**). To elucidate whether the decrease in NK cells reflected altered tissue egress or peripheral maintenance, we next assessed possible changes of vascular versus parenchymal NK cells in different tissues using intravenous injection of CD45-PE to label intravascular NK cells. As observed in in IL-15rα^flox^ Cdh5-iCre mice (**Supplementary Figure S5B**), the abundance of total NK cells in the bone marrow of IL-15^flox^ Cdh5-iCre mice was unchanged (**Figure 5F**). However, vascular PE-labelled NK cells, and among them specifically mature CD11b^+^CD27^-^ NK cells, were reduced while their parenchymal counterparts remained unaffected (**Figures 5G, H**, **Supplementary Figures S5I, J**). In the spleen, parenchymal NK cells were more pronouncedly decreased in the absence of endothelial IL-15 compared to vascular NK cells (**Figure 5H**, **Supplementary Figure S5K**). Similarly, total and parenchymal NK cells in the liver of IL-15^flox^ Cdh5-iCre mice were significantly reduced compared to littermate controls (**Figure 5I**, **Supplementary Figure S5L**). A not statistically significant reduction was observed for vascular NK cells in spleen and liver. These results indicate that endothelial IL-15 deletion specifically supports mature NK cell maintenance in the circulation, in the (peri-)vascular niche of the bone marrow and in highly vascularized organs such as the spleen and liver.

In summary, endothelial IL-15/IL-15rα deletion had a pronounced impact on peripheral mature NK cells, while central memory CD8^+^ T cell abundance was unaffected.

## 4 Discussion

IL-15 is a critical cytokine for the survival and function of several lymphocyte subsets, including memory CD8^+^ T cells, NK cells and iNKT cells (6, 47–49). While it is well established that IL-15 is primarily presented *in trans* by cells expressing IL-15Rα (7, 50), the precise cellular sources and mechanisms of this transpresentation in the BM remain incompletely understood. In this study, we investigated the contribution of BM stromal cells to IL-15/IL-15Rα-mediated support of lymphocyte populations, with a focus on dissecting the cellular and spatial heterogeneity of this system. We reveal that distinct stromal cell populations exhibit highly variable expression of IL-15 and IL-15Rα, and that different lymphocyte subsets rely on specific stromal niches for IL-15Rα-dependent maintenance and survival.

We and others have previously shown that BM stromal cells, particularly MSCs, are capable of producing IL-15 (51) and that stromal IL-15 contributes to the maintenance of memory CD8^+^ T cells and developing NK cells (23). However, it remains unclear whether these cells are capable of transpresenting IL-15 via IL-15Rα, or whether alternative mechanisms of IL-15 delivery are used. Analysis of scRNA-seq data demonstrated a striking decoupling of IL-15 and IL-15Rα mRNA levels among BM stromal subsets (**Figure 1**). This would suggest an unevenly distributed capacity for IL-15 transpresentation across the stromal compartment, theoretically requiring intercellular cooperation between IL-15^+^ and IL-15Rα^+^ cells. While transpresentation is likely the primary mode of IL-15 signaling, previous studies also support non-classical or uncoupled mechanisms of IL-15activity, e.g. in the context of tumor growth where IL-15Rα can mediate autonomous survival signaling (52). Likewise, there is some evidence of *in cis* signaling functions of the IL-15Rα (53), e.g. by supporting stromal function and bone mineralization (54, 55). Similarly, IL-15Rα has been implicated in IL-15-independent *in cis* signaling of IL-17-producing γδ T cells, suggesting functional plasticity of the receptor (56). Additionally, both IL-15 and IL-15Rα are heavily post-transcriptionally regulated, complicating the interpretation of RNA expression data (15, 57). Lepr^+^ MSCs, a known source of IL-15, showed negligible IL-15Rα expression by scRNA-seq, while other subsets such as osteo-lineage MSCs and pericytes had a higher IL-15Rα/IL-15 ratio. However, scRNA-seq very likely underestimates low-abundance transcripts like IL-15Rα (38), since IL-15-GFP expression still correlated with IL-15Rα mRNA abundance in stromal cells. We further demonstrate that Lepr^+^ MSCs, despite low IL-15Rα mRNA expression, contribute to the maintenance of central memory (T_CM_) CD8^+^ T cells via IL-15 transpresentation (**Figure 2**). Conditional deletion of either IL-15 or IL-15Rα in Lepr^+^ MSCs led to a comparable slight reduction in BM-resident T_CM_ cells under steady state conditions (23), consistent with the requirement of both components for transpresentation. Fate mapping with IL-7-Cre Rosa26-YFP mice further revealed that a subset of Lepr^+^ MSCs co-expressed IL-7, identifying them as a potential double source of IL-7 and IL-15 signals essential for T_CM_ survival. Prior studies have emphasized the complementary roles of IL-7 and IL-15 in maintaining CD8^+^ T cell memory, and different memory T cell subsets show varying degrees of IL-7 dependency (40, 58). Notably, the effect of Lepr/IL-7-mediated IL-15Rα deletion was specific to T_CM_ cells and spared tissue-resident memory (T_RM_) cells and NKT cells, supporting the notion of a specific IL-7/IL-15 codependency niche (48). Interestingly, the reduction of T_CM_ cells upon IL-15Rα deletion in IL-7^+^ MSCs also led to a slight, albeit not statistically significant, increase in effector memory (T_EM_) cells, potentially due to niche competition and the reallocation of resources. However, the CD8 T_CM_ phenotype was subtle under steady-state conditions, especially with regard to absolute quantification. Since the reduction of bone marrow CD8 T_CM_ in an Lepr-Cre induced IL-15 deletion model has been shown to be age-dependent (23), suggesting that the subtle phenotype observed here in younger adult mice (8–16 weeks) may become more pronounced in older animals. Nonetheless, further studies under conditions of immune activation, like infection or vaccination, are required to determine whether these subtle steady-state alterations have functional consequences or remain without biological relevance.

While Lepr^+^ MSCs predominantly support T_CM_ cells, we found that endosteal stromal cells targeted by Osx-Cre additionally maintain CD8 T_RM_ and NKT cells through IL-15 transpresentation (**Figure 3**). Deletion of IL-15Rα in Osx^+^ cells resulted in a marked reduction of these lymphocyte populations, indicating that endosteal niches are functionally distinct from other BM stromal niches. Surprisingly, deletion of IL-15Rα in mesenchymal stromal cells using Prx1-Cre had only modest effects on CD8^+^ T cells, despite targeting a diverse range of stromal populations (**Figure 4**). One possible explanation for this attenuated phenotype is the presence of Prx1-targeted stromal subsets, such as IL-15rα^+^ IL-15^-^ pericytes, that may act as local cytokine “scavengers”. Similar to CD25-mediated IL-2 scavenging by regulatory T cells, IL-15Rα-expressing stromal cells may buffer IL-15 availability, thus modulating the cytokine landscape. This model is supported by literature describing anti-inflammatory functions of (soluble) IL-15Rα and its role in fine-tuning cytokine responses (42, 43). While this hypothesis remains to be functionally tested, the observed expression pattern of IL-15 and IL-15rα in bone marrow pericytes is consistent with a potential modulatory role of these cells in local IL-15 availability. Such an effect could contribute to the attenuated phenotype observed upon IL-15rα deletion in Prx1-Cre mice.

Our data further implicate endothelial cells in the support of NK cells through IL-15 transpresentation (**Figure 5**). Deletion of IL-15Rα in endothelial cells impaired NK cell maintenance in the periphery, yet had a minor effect on CD8^+^ T cells, which is surprising since endothelial IL-15 deletion has been shown to affect CD8^+^ T cells as well (23). This aligns with previous work suggesting that soluble IL-15 may be sufficient to expand memory CD8^+^ T cells, which are less reliant on cell-bound IL-15Rα transpresentation than NK cells, highlighting differential sensitivity among lymphocyte subsets (59, 60). IL-15 has also been shown to enhance endothelial-NK cell adhesion via LFA-1/ICAM-1 (61) and endothelial IL-15 can influence lymphocyte trans-endothelial migration (45). Along these lines, we also observed subtle changes in adhesion molecule expression on both NK cells and splenic endothelial cells, suggesting a potential role of endothelial IL-15Rα in NK-endothelial cellular interactions.

There is increasing evidence that IL-15 functions in both a homeostatic and an inflammatory mode, which are shaped by differences in dose, cellular source, and context. We showed that, under steady-state conditions, stromal and endothelial cells provide low-level, spatially restricted IL-15 transpresentation that moderately supports NK and CD8^+^ T-cells, whereas during acute activation dendritic cells and monocytes have been shown to rapidly upregulate IL-15 to drive effector responses (5, 7, 62). In line with this model, we have previously shown that NK cells from stromal IL-15-deficient mice retain normal per-cell cytokine production (23), suggesting that stromal IL-15 mainly calibrates homeostatic development rather than acute effector function. Although we would not expect stromal IL-15 deletion to impair early responses during acute infection, future studies will be required to determine whether stromal IL-15 influences immune function in chronic inflammatory settings, including tumor growth, tissue rechallenge, or sustained antigen exposure, where prolonged niche interactions and long-term memory formation may become limiting factors.

Together, our findings support a model in which IL-15 transpresentation is compartmentalized within specialized stromal niches of the BM and the periphery. Not all stromal cells support all lymphocytes equally. Rather, specific subsets are tailored to deliver IL-15 or IL-15Rα/IL-15 to distinct immune cell populations. The observed effects remained moderate, indicating that other cellular sources can compensate for the loss of stromal IL-15/IL-15Rα. This niche specialization ensures the maintenance of immune cell diversity and allows for context-specific modulation of cytokine signaling. Thus, our work highlights the importance of accounting for stromal cell heterogeneity in studies of immune regulation, with implications for regenerative medicine, autoimmune disease research and cancer.

## Data Availability

The original scRNA-seq data generated in the present study have been deposited on the Open Science Framework platform (accessible via https://osf.io/b3tex/overview?view_only=634be02df77449ffbad7b1b41de484a9). Re-analyzed bone marrow datasets from previously published studies were derived from Gene Expression Omnibus (accession numbers GSE156635, GSE122467, GSE108892, GSE128423 and GSE273212).

## References

[1] Di Rosa F . Maintenance of memory T cells in the bone marrow: survival or homeostatic proliferation? Nat Rev Immunol. (2016) 16:271–1. doi: 10.1038/nri.2016.31, PMID: 26996200

[2] Yu J Freud AG Caligiuri MA . Location and cellular stages of NK cell development. Trends Immunol. (2013) 34:573–82. doi: 10.1016/j.it.2013.07.005, PMID: 24055329 PMC3852183

[3] Méndez-Ferrer S Michurina TV Ferraro F Mazloom AR Macarthur BD Lira SA . Mesenchymal and haematopoietic stem cells form a unique bone marrow niche. Nature. (2010) 466:829–34. doi: 10.1038/nature09262, PMID: 20703299 PMC3146551

[4] Lodolce JP Boone DL Chai S Swain RE Dassopoulos T Trettin S . IL-15 receptor maintains lymphoid homeostasis by supporting lymphocyte homing and proliferation. Immunity. (1998) 9:669–76. doi: 10.1016/S1074-7613(00)80664-0, PMID: 9846488

[5] Mortier E Advincula R Kim L Chmura S Barrera J Reizis B . Macrophage- and dendritic-cell-derived interleukin-15 receptor alpha supports homeostasis of distinct CD8+ T cell subsets. Immunity. (2009) 31:811–22. doi: 10.1016/J.IMMUNI.2009.09.017, PMID: 19913445

[6] Kennedy MK Glaccum M Brown SN Butz EA Viney JL Embers M . Reversible defects in natural killer and memory CD8 T cell lineages in interleukin 15-deficient mice. J Exp Med. (2000) 191:771–80. doi: 10.1084/JEM.191.5.771, PMID: 10704459 PMC2195858

[7] Dubois S Mariner J Waldmann TA Tagaya Y . IL-15Ralpha recycles and presents IL-15 In trans to neighboring cells. Immunity. (2002) 17:537–47. doi: 10.1016/S1074-7613(02)00429-6, PMID: 12433361

[8] Stonier SW Schluns KS . Trans-presentation: a novel mechanism regulating IL-15 delivery and responses. Immunol Lett. (2010) 127:85–92. doi: 10.1016/j.imlet.2009.09.009, PMID: 19818367 PMC2808451

[9] Santana Carrero RM Beceren-Braun F Rivas SC Hegde SM Gangadharan A Plote D . IL-15 is a component of the inflammatory milieu in the tumor microenvironment promoting antitumor responses. Proc Natl Acad Sci. (2019) 116:599–608. doi: 10.1073/pnas.1814642116, PMID: 30587590 PMC6329954

[10] Meazza R Gaggero A Neglia F Basso S Sforzini S Pereno R . Expression of two interleukin-15 mRNA isoforms in human tumors does not correlate with secretion: role of different signal peptides. Eur J Immunol. (1997) 27:1049–54. doi: 10.1002/eji.1830270502, PMID: 9174591

[11] Bamford RN DeFilippis AP Azimi N Kurys G Waldmann TA . The 5’ untranslated region, signal peptide, and the coding sequence of the carboxyl terminus of IL-15 participate in its multifaceted translational control. J Immunol. (1998) 160:4418–26. doi: 10.4049/jimmunol.160.9.4418, PMID: 9574546

[12] Wang X Zhao X-Y . Transcription Factors Associated With IL-15 Cytokine Signaling During NK Cell Development. Front Immunol. (2021) 12:610789. doi: 10.3389/fimmu.2021.610789, PMID: 33815365 PMC8013977

[13] Tagaya Y Kurys G Thies TA Losi JM Azimi N Hanover JA . Generation of secretable and nonsecretable interleukin 15 isoforms through alternate usage of signal peptides. Proc Natl Acad Sci Unite States America. (1997) 94:14444–9. doi: 10.1073/PNAS.94.26.14444, PMID: 9405632 PMC25016

[14] Wu X Pan W Stone KP Zhang Y Hsuchou H Kastin AJ . Expression and signaling of novel IL15Rα splicing variants in cerebral endothelial cells of the blood-brain barrier. J Neurochem. (2010) 114:122–9. doi: 10.1111/j.1471-4159.2010.06729.x, PMID: 20374432 PMC2905142

[15] Tamzalit F Barbieux I Plet A Heim J Nedellec S Morisseau S . IL-15.IL-15Rα complex shedding following trans-presentation is essential for the survival of IL-15 responding NK and T cells. Proc Natl Acad Sci. (2014) 111:8565–70. doi: 10.1073/pnas.1405514111, PMID: 24912180 PMC4060713

[16] Tao H Li L Liao N-S Schluns KS Luckhart S Sleasman JW . Thymic Epithelial Cell-Derived IL-15 and IL-15 Receptor α Chain Foster Local Environment for Type 1 Innate Like T Cell Development. Front Immunol. (2021) 12:623280. doi: 10.3389/fimmu.2021.623280, PMID: 33732245 PMC7957058

[17] Castillo EF Acero LF Stonier SW Zhou D Schluns KS . Thymic and peripheral microenvironments differentially mediate development and maturation of iNKT cells by IL-15 transpresentation. Blood. (2010) 116:2494–503. doi: 10.1182/blood-2010-03-277103, PMID: 20581314 PMC2953886

[18] Huntington ND Legrand N Alves NL Jaron B Weijer K Plet A . IL-15 trans-presentation promotes human NK cell development and differentiation *in vivo*. J Exp Med. (2009) 206:25–34. doi: 10.1084/jem.20082013, PMID: 19103877 PMC2626663

[19] Huntington ND . The unconventional expression of IL-15 and its role in NK cell homeostasis. Immunol Cell Biol. (2014) 92:210–3. doi: 10.1038/icb.2014.1, PMID: 24492800

[20] Burkett PR Koka R Chien M Chai S Chan F Ma A . IL-15Rα expression on CD8+ T cells is dispensable for T cell memory. Proc Natl Acad Sci. (2003) 100:4724–9. doi: 10.1073/pnas.0737048100, PMID: 12671073 PMC153623

[21] Kokaji AI Hockley DL Kane KP . IL-15 transpresentation augments CD8+ T cell activation and is required for optimal recall responses by central memory CD8+ T cells. J Immunol. (2008) 180:4391–401. doi: 10.4049/jimmunol.180.7.4391, PMID: 18354159

[22] Castillo EF Schluns KS . Regulating the immune system via IL-15 transpresentation. Cytokine. (2012) 59:479–90. doi: 10.1016/j.cyto.2012.06.017, PMID: 22795955 PMC3422378

[23] Stecher C Bischl R Schmid-Böse A Ferstl S Potzmann E Frank M . Heterogeneity of IL-15-expressing mesenchymal stromal cells controls natural killer cell development and immune cell homeostasis. Nat Commun. (2025) 16:5949. doi: 10.1038/s41467-025-61231-0, PMID: 40595636 PMC12218584

[24] Liou Y-H Wang S-W Chang C-L Huang P-L Hou M-S Lai Y-G . Adipocyte IL-15 Regulates Local and Systemic NK Cell Development. J Immunol. (2014) 193:1747–58. doi: 10.4049/JIMMUNOL.1400868/-/DCSUPPLEMENTAL, PMID: 25009203

[25] Repass JF Laurent MN Carter C Reizis B Bedford MT Cardenas K . IL7-hCD25 and IL7-Cre BAC transgenic mouse lines: new tools for analysis of IL-7 expressing cells. Genesis. (2009) 47:281–7. doi: 10.1002/DVG.20497, PMID: 19263498

[26] Sörensen I Adams RH Gossler A . DLL1-mediated Notch activation regulates endothelial identity in mouse fetal arteries. Blood. (2009) 113:5680–8. doi: 10.1182/blood-2008-08-174508, PMID: 19144989

[27] Greenbaum A Hsu YMS Day RB Schuettpelz LG Christopher MJ Borgerding JN . CXCL12 in early mesenchymal progenitors is required for haematopoietic stem-cell maintenance. Nature. (2013) 495:227–30. doi: 10.1038/NATURE11926, PMID: 23434756 PMC3600148

[28] Ding L Morrison SJ . Haematopoietic stem cells and early lymphoid progenitors occupy distinct bone marrow niches. Nature. (2013) 495:231–5. doi: 10.1038/nature11885, PMID: 23434755 PMC3600153

[29] Zhou BO Yue R Murphy MM Peyer JG Morrison SJ . Leptin Receptor-expressing mesenchymal stromal cells represent the main source of bone formed by adult bone marrow. Cell Stem Cell. (2014) 15:154. doi: 10.1016/J.STEM.2014.06.008, PMID: 24953181 PMC4127103

[30] Rodda SJ McMahon AP . Distinct roles for Hedgehog and canonical Wnt signaling in specification,differentiation and maintenance of osteoblast progenitors. Development. (2006) 133:3231–44. doi: 10.1242/dev.02480, PMID: 16854976

[31] Marsh S Salmon M Hoffman P kew24 . samuel-marsh/scCustomize. (2024). doi: 10.5281/zenodo.14529706. Genéve, Switzerland: European Organization for Nuclear Research (CERN).

[32] Baryawno N Przybylski D Kowalczyk MS Kfoury Y Severe N Gustafsson K . A Cellular Taxonomy of the Bone Marrow Stroma in Homeostasis and Leukemia. Cell. (2019) 177:1915–1932.e16. doi: 10.1016/J.CELL.2019.04.040, PMID: 31130381 PMC6570562

[33] Tikhonova AN Dolgalev I Hu H Sivaraj KK Hoxha E Cuesta-Domínguez Á . The bone marrow microenvironment at single-cell resolution. Nature. (2019) 569:222–8. doi: 10.1038/S41586-019-1104-8, PMID: 30971824 PMC6607432

[34] Baccin C Al-Sabah J Velten L Helbling PM Grünschläger F Hernández-Malmierca P . Combined single-cell and spatial transcriptomics reveal the molecular, cellular and spatial bone marrow niche organization. Nat Cell Biol. (2019) 22:38–48. doi: 10.1038/s41556-019-0439-6, PMID: 31871321 PMC7610809

[35] Sivaraj KK Jeong H-W Dharmalingam B Zeuschner D Adams S Potente M . Regional specialization and fate specification of bone stromal cells in skeletal development. Cell Rep. (2021) 36:109352. doi: 10.1016/j.celrep.2021.109352, PMID: 34260921 PMC8293626

[36] Anderson KG Mayer-Barber K Sung H Beura L James BR Taylor JJ . Intravascular staining for discrimination of vascular and tissue leukocytes. Nat Protoc. (2014) 9:209–22. doi: 10.1038/nprot.2014.005, PMID: 24385150 PMC4428344

[37] Herndler-Brandstetter D Ishigame H Shinnakasu R Plajer V Stecher C Zhao J . KLRG1+ Effector CD8+ T Cells Lose KLRG1, Differentiate into All Memory T Cell Lineages, and Convey Enhanced Protective Immunity. Immunity. (2018) 48:716–29.e8. doi: 10.1016/j.immuni.2018.03.015, PMID: 29625895 PMC6465538

[38] Hicks SC Townes FW Teng M Irizarry RA . Missing data and technical variability in single-cell RNA-sequencing experiments. Biostatistics. (2018) 19:562–78. doi: 10.1093/biostatistics/kxx053, PMID: 29121214 PMC6215955

[39] Nanjappa SG Walent JH Morre M Suresh M . Effects of IL-7 on memory CD8+ T cell homeostasis are influenced by the timing of therapy in mice. J Clin Invest. (2008) 118:1027–39. doi: 10.1172/JCI32020, PMID: 18246202 PMC2214844

[40] Rubinstein MP Lind NA Purton JF Filippou P Best JA McGhee PA . IL-7 and IL-15 differentially regulate CD8+ T-cell subsets during contraction of the immune response. Blood. (2008) 112:3704–12. doi: 10.1182/blood-2008-06-160945, PMID: 18689546 PMC2572798

[41] Davey RA Clarke MV Sastra S Skinner JP Chiang C Anderson PH . Decreased body weight in young Osterix-Cre transgenic mice results in delayed cortical bone expansion and accrual. Transgenic Res. (2012) 21:885–93. doi: 10.1007/s11248-011-9581-z, PMID: 22160436

[42] Ruchatz H Leung BP Wei X McInnes IB Liew FY . Soluble IL-15 Receptor α-Chain Administration Prevents Murine Collagen-Induced Arthritis: A Role for IL-15 in Development of Antigen-Induced Immunopathology1. J Immunol. (1998) 160:5654–60. doi: 10.4049/jimmunol.160.11.5654, PMID: 9605172

[43] Bouchaud G Gehrke S Krieg C Kolios A Hafner J Navarini AA . Epidermal IL-15Rα acts as an endogenous antagonist of psoriasiform inflammation in mouse and man. J Exp Med. (2013) 210:2105–17. doi: 10.1084/jem.20130291, PMID: 24019554 PMC3782049

[44] Chen J Shi Y Regan J Karuppaiah K Ornitz DM Long F . Osx-Cre targets multiple cell types besides osteoblast lineage in postnatal mice. PloS One. (2014) 9:e85161. doi: 10.1371/journal.pone.0085161, PMID: 24454809 PMC3893188

[45] Oppenheimer-Marks N Brezinschek RI Mohamadzadeh M Vita R Lipsky PE . Interleukin 15 is produced by endothelial cells and increases the transendothelial migration of T cells *In vitro* and in the SCID mouse-human rheumatoid arthritis model *In vivo*. J Clin Invest. (1998) 101:1261. doi: 10.1172/JCI1986, PMID: 9502767 PMC508680

[46] Mullan CW Summer L Lopez-Giraldez F Tobiasova Z Manes TD Yasothan S . IL-1β Induces Human Endothelial Surface Expression of IL-15 by Relieving let-7c-3p Suppression of Protein Translation. J Immunol. (2024) 213:1338–48. doi: 10.4049/jimmunol.2400331, PMID: 39302113 PMC11493510

[47] Judge AD Zhang X Fujii H Surh CD Sprent J . Interleukin 15 Controls both Proliferation and Survival of a Subset of Memory-Phenotype CD8+ T Cells. J Exp Med. (2002) 196:935–46. doi: 10.1084/jem.20020772, PMID: 12370255 PMC2194030

[48] Matsuda JL Gapin L Sidobre S Kieper WC Tan JT Ceredig R . Homeostasis of V alpha 14i NKT cells. Nat Immunol. (2002) 3:966–74. doi: 10.1038/ni837, PMID: 12244311

[49] Burkett PR Koka R Chien M Chai S Boone DL Ma A . Coordinate Expression and Trans Presentation of Interleukin (IL)-15Rα and IL-15 Supports Natural Killer Cell and Memory CD8+ T Cell Homeostasis. J Exp Med. (2004) 200:825–34. doi: 10.1084/jem.20041389, PMID: 15452177 PMC2213280

[50] Burkett PR Koka R Chien M Chai S Boone DL Ma A . Coordinate expression and trans presentation of interleukin (IL)-15Ralpha and IL-15 supports natural killer cell and memory CD8+ T cell homeostasis. J Exp Med. (2004) 200:825–34. doi: 10.1084/jem.20041389, PMID: 15452177 PMC2213280

[51] Cui G Hara T Simmons S Wagatsuma K Abe A Miyachi H . Characterization of the IL-15 niche in primary and secondary lymphoid organs *in vivo*. Proc Natl Acad Sci. (2014) 111:1915–20. doi: 10.1073/pnas.1318281111, PMID: 24449915 PMC3918838

[52] Marra P Mathew S Grigoriadis A Wu Y Kyle-Cezar F Watkins J . IL15RA Drives Antagonistic Mechanisms of Cancer Development and Immune Control in Lymphocyte-Enriched Triple-Negative Breast Cancers. Cancer Res. (2014) 74:4908–21. doi: 10.1158/0008-5472.CAN-14-0637, PMID: 24980552

[53] Wu Z Xue H-H Bernard J Zeng R Issakov D Bollenbacher-Reilley J . The IL-15 receptor α chain cytoplasmic domain is critical for normal IL-15Rα function but is not required for trans-presentation. Blood. (2008) 112:4411–9. doi: 10.1182/blood-2007-03-080697, PMID: 18796634 PMC2597117

[54] Loro E Ramaswamy G Chandra A Tseng W-J Mishra MK Shore EM . IL15RA is required for osteoblast function and bone mineralization. Bone. (2017) 103:20–30. doi: 10.1016/j.bone.2017.06.003, PMID: 28602725 PMC5598756

[55] Kornsuthisopon C Manokawinchoke J Sonpoung O Osathanon T Damrongsri D . Interleukin 15 participates in Jagged1-induced mineralization in human dental pulp cells. Arch Oral Biol. (2021) 128:105163. doi: 10.1016/j.archoralbio.2021.105163, PMID: 34058721

[56] Colpitts SL Puddington L Lefrançois L . IL-15 receptor α signaling constrains the development of IL-17-producing γδ T cells. Proc Natl Acad Sci U.S.A. (2015) 112:9692–7. doi: 10.1073/pnas.1420741112, PMID: 26195801 PMC4534247

[57] Bamford RN Battiata AP Waldmann TA . IL-15: the role of translational regulation in their expression. J Leukoc Biol. (1996) 59:476–80. doi: 10.1002/jlb.59.4.476, PMID: 8613692

[58] Cui W Kaech SM . Generation of effector CD8+ T cells and their conversion to memory T cells. Immunol Rev. (2010) 236:151–66. doi: 10.1111/j.1600-065X.2010.00926.x, PMID: 20636815 PMC4380273

[59] Berger A Colpitts SJ Seabrook MSS Furlonger CL Bendix MB Moreau JM . Interleukin-15 in cancer immunotherapy: IL-15 receptor complex versus soluble IL-15 in a cancer cell-delivered murine leukemia model. J ImmunoTher Cancer. (2019) 7:355. doi: 10.1186/S40425-019-0777-8, PMID: 31856922 PMC6924073

[60] Ota N Takase M Uchiyama H Olsen SK Kanagawa O . No requirement of trans presentations of IL-15 for human CD8 T cell proliferation. J Immunol. (2010) 185:6041–8. doi: 10.4049/JIMMUNOL.0901834, PMID: 20926799

[61] Allavena P Giardina G Bianchi G Mantovani A . IL-15 is chemotactic for natural killer cells and stimulates their adhesion to vascular endothelium. J Leukoc Biol. (1997) 61:729–35. doi: 10.1002/jlb.61.6.729, PMID: 9201264

[62] Mortier E Woo T Advincula R Gozalo S Ma A . IL-15Rα chaperones IL-15 to stable dendritic cell membrane complexes that activate NK cells via trans presentation. J Exp Med. (2008) 205:1213–25. doi: 10.1084/JEM.20071913, PMID: 18458113 PMC2373851

