# Supplementary material for: Transpresentation of interleukin 15 by stromal cell subsets regulates immune cell homeostasis
Source: Front Immunol. 2026 Jan 16;16:1673309. doi: 10.3389/fimmu.2025.1673309 (PMC12855473; doi:10.3389/fimmu.2025.1673309)
Supplement: Supplementary file 1 [file DataSheet1.pdf]

Supplementary Material

1.1 Supplementary Figures

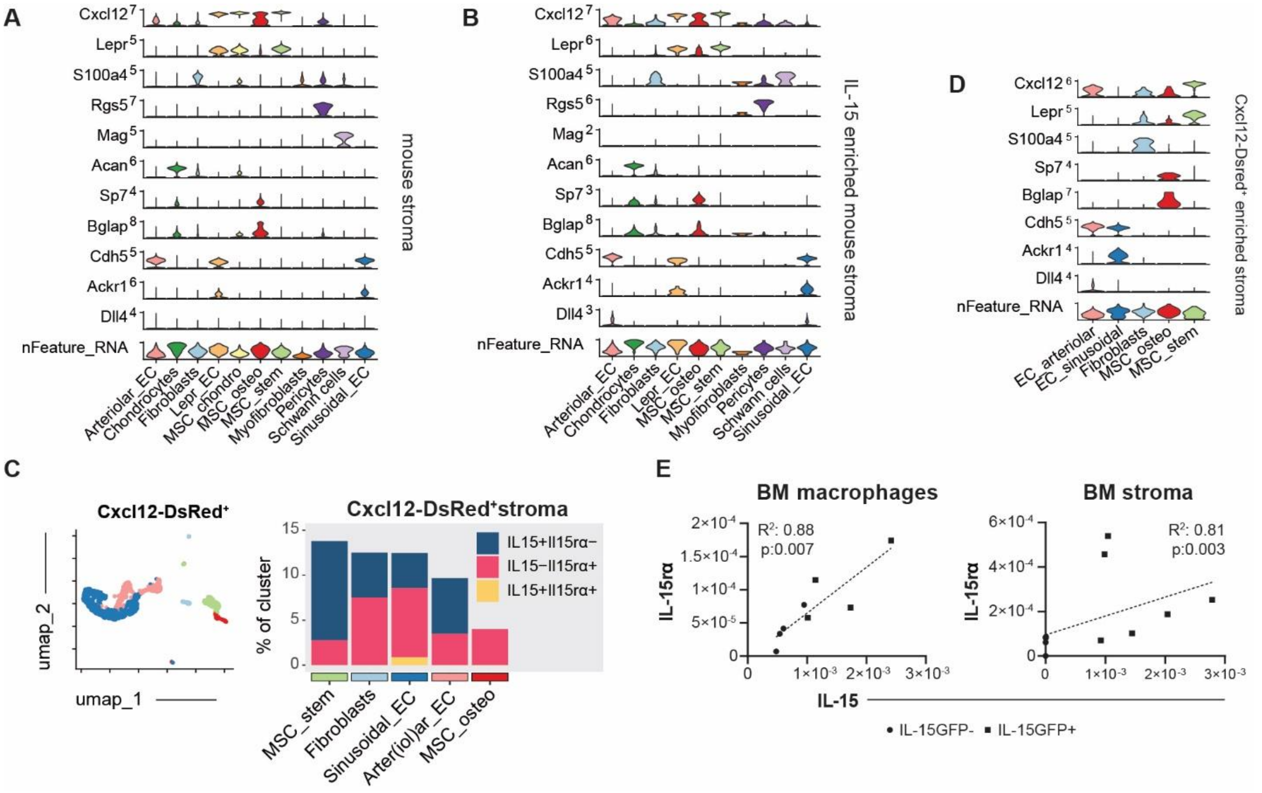

Supplementary Figure 1. Stromal cell clustering of public and own scRNA-seq datasets, related to Figure 1.

(A) Stacked Violin Plots showing mRNA expression of the indicated mouse bone marrow cluster defining genes for each cell cluster, related to Figure 1A. **(B)** Stacked Violin Plots showing mRNA expression of the indicated genes for each cell cluster from IL-15^{GFP} enriched mouse bone marrow stroma, related to Figure 1B. **(C)** Umap plot showing stromal cell clusters from scRNA-sequencing of DsRed^{+} stromal cells sorted from Cxcl12-DsRed$^{+/-}$ reporter mice (left) and relative quantification of IL-15- versus IL-15Rα-expressing cells. **(D)** Stacked Violin Plots showing mRNA expression of the indicated genes for each cluster from Cxcl12-DsRed^{+} stroma in D. **(E)** Correlation plot showing quantification of IL-15 and IL-15Rα via qPCR (normalized to beta-Actin) in IL-15GFP^{+} and IL-15GFP^{-} sorted F4/80^{+} macrophages (left) and CD31^{-}Ter119^{-}CD45^{-} stromal cells (right). Numbers indicate R^2 and *P* values calculated from two-tailed Spearman's rank correlations.

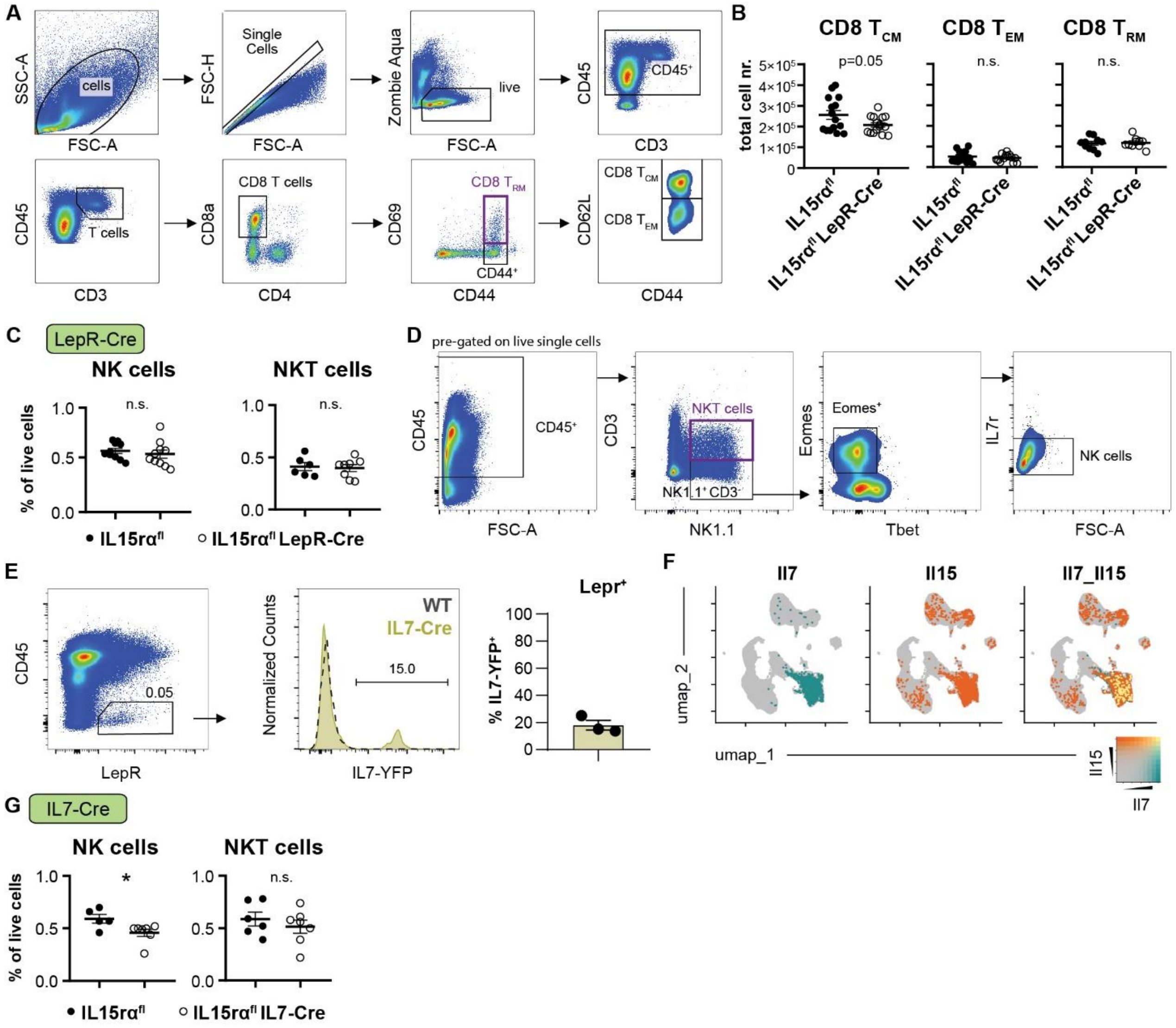

Supplementary Figure 2. Abundance of cell types in Lepr-Cre and IL-7-Cre mice, related to Figure 2.

(A) Flow cytometry gating strategy for the quantification of central memory (T_{CM}), resident memory (T^{RM}) and effector memory (T^{EM}) $CD8^+$ T cells. **(B)** Total cell numbers in the bone marrow of IL 15R$\alpha^{flox/flox}$ and IL 15R$\alpha^{flox/flox}$ Lepr-Cre mice. **(C)** Relative abundance of ($NK1.1^+CD3^-Eomes^+$) NK cells and ($CD3^+NK1.1^+$) NKT cells in the bone marrow of IL-15R$\alpha^{flox/flox}$ Lepr-Cre mice and IL-15R$\alpha^{flox/flox}$ controls. **(D)** Flow cytometry gating strategy for the quantification of NK cells and NKT cells. **(E)** Representative flow cytometry plot and quantification of IL-7-YFP expression among $Lepr^+$ bone marrow stromal cells. **(F)** Feature plots showing separate and combined IL-7 and IL-15 expression in the mouse bone marrow stroma scRNA-seq dataset from Figure 1A. **(G)** Relative abundance of ($NK1.1^+CD3^-Eomes^+$) NK cells and ($CD3^+NK1.1^+$) NKT cells in the bone marrow of IL-15R$\alpha^{flox/flox}$ IL-7-Cre mice and IL-15R$\alpha^{flox/flox}$ controls. Asterisks above the dot plots indicate *P* values from unpaired two-tailed Student's *t* tests. *$P < 0.05$, n.s. $P > 0.05$.

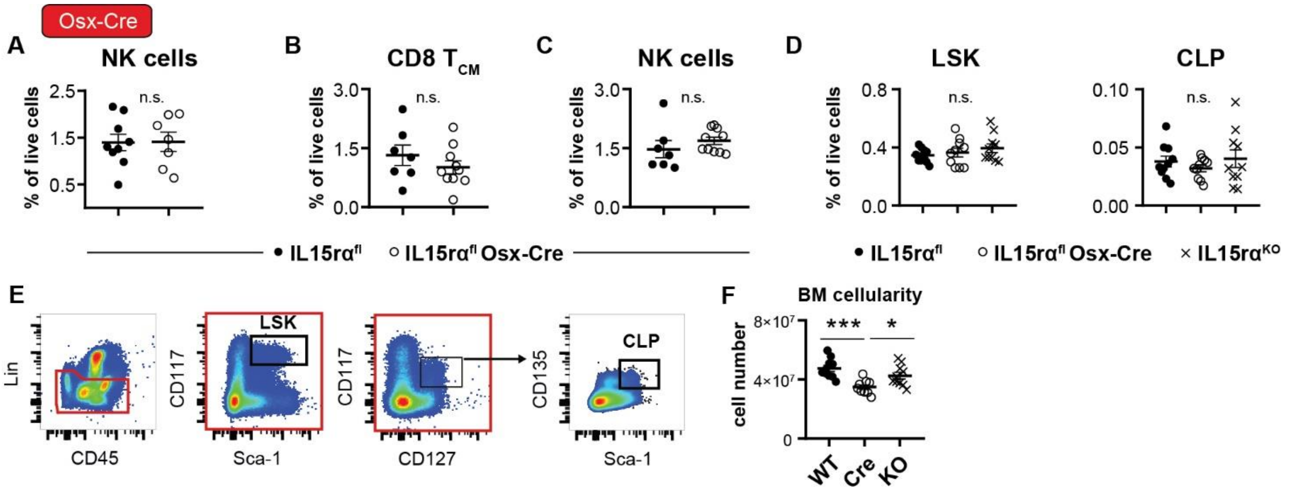

Supplementary Figure 3. Abundance of IL-15-dependent immune cell types in different organs of IL-15Rα^{flox} Osx-Cre mice, related to Figure 3.

(A) Relative abundance of NK cells (CD3$^-$NK1.1$^+$CD49b$^+$CD127$^-$) in the spleen of IL-15R$\alpha^{flox/flox}$ Osx-Cre mice compared to IL-15R$\alpha^{flox/flox}$ littermates. **(B)** Relative abundance of central memory CD8$^+$ T cells (CD8 T_{CM}) in the peripheral blood of IL-15R$\alpha^{flox/flox}$ Osx-Cre mice compared to Cre negative littermates. **(C)** Relative abundance of NK cells (CD3$^-$NK1.1$^+$CD49b$^+$CD127$^-$) in the peripheral blood of IL-15R$\alpha^{flox/flox}$ Osx-Cre mice compared to Cre negative littermates. Quantification **(D)** and gating strategy **(E)** of hematopoietic stem cells (LSK) and common lymphoid progenitor cells (CLP) in the bone marrow of IL-15R$\alpha^{flox/flox}$ Osx-Cre mice and IL-15R$\alpha^{flox/flox}$ littermates compared to IL-15R$\alpha^{KO/KO}$ total knockout mice. The lineage cocktail (Lin) contained antibodies against CD3, CD19, NK1.1, Gr-1 and Ter119. (**F**) Quantification of total bone marrow cellularity in IL-15Rαflox/flox (WT) mice, IL-15Rαflox/flox Osx-Cre littermates (Cre) and IL-15RαKO/KO mice. Asterisks above the dot plots indicate P values from unpaired two-tailed Student's t tests or Tukey's multiple comparisons test after One-Way ANOVA. *$P < 0.05$, **$P < 0.01$, ***$P < 0.001$, ****$P < 0.0001$, n.s. $P > 0.05$.

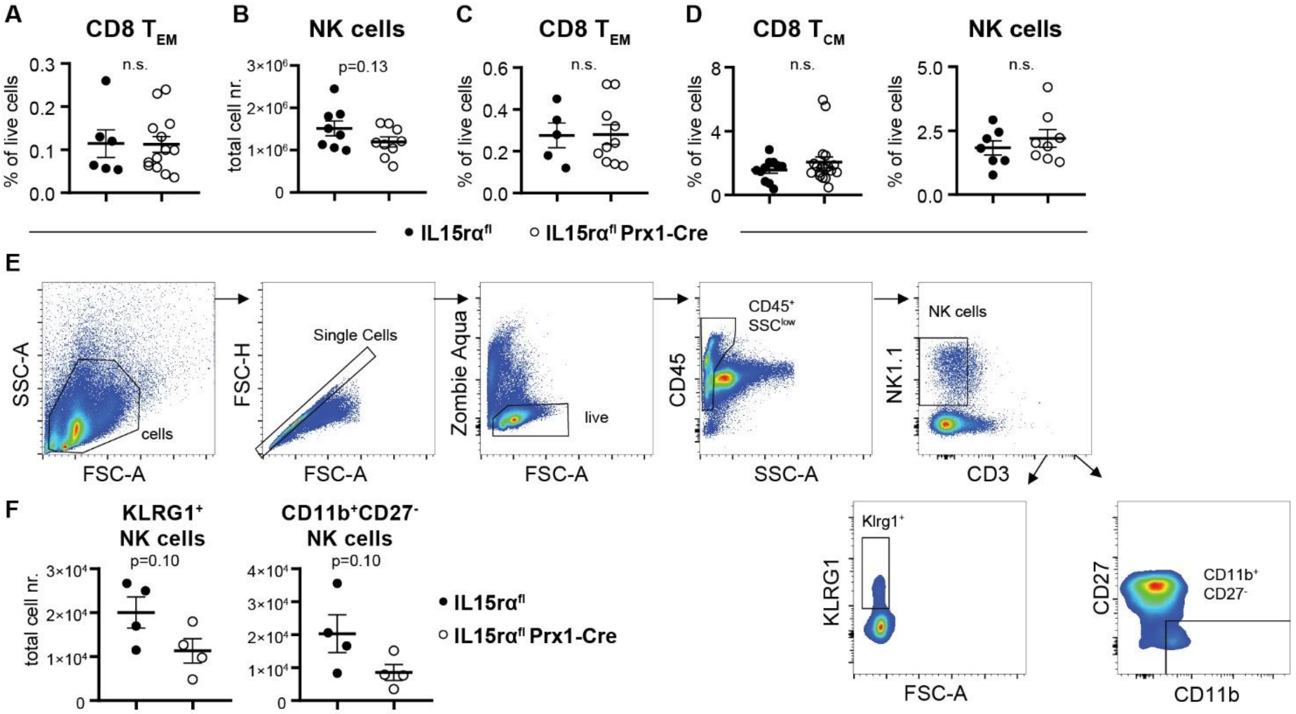

Supplementary Figure 4. Abundance of IL-15-dependent immune cell types in different organs of IL-15Rα^{flox} Prx1-Cre mice, related to Figure 4.

(A) Relative abundance of effector/memory CD8^{+} T cells (CD8 T_{EM}) in the bone marrow of IL-15Rα$^{flox/flox}$ Prx1-Cre mice compared to Cre negative littermates. **(B)** Absolute quantification of NK cell numbers in the spleen of IL-15Rα$^{flox/flox}$ and IL-15Rα$^{flox/flox}$ Prx1-Cre mice. **(C)** Relative abundance of CD8 T_{EM} in the spleen of IL-15Rα$^{flox/flox}$ Prx1-Cre mice compared to Cre negative littermates. **(D)** Quantification of central memory CD8^{+} T cells (CD8 T_{CM}) and NK cells (CD3^{-}NK1.1^{+}CD49b^{+}CD127^{-}) in the blood of IL-15Rα$^{flox/flox}$ and IL-15Rα$^{flox/flox}$ Prx1-Cre littermates. **(E)** Flow cytometric gating strategy for NK cell maturation markers. (**F**) Absolute quantification of Klrg1 expressing NK cells (left) and mature CD11b^{+}CD27^{-} NK cells (right) in the bone marrow of IL-15Rα$^{flox/flox}$ and IL-15Rα$^{flox/flox}$ Prx1-Cre mice. Numbers above the dot plots indicate *P* values from unpaired two-tailed Student’s *t* tests.

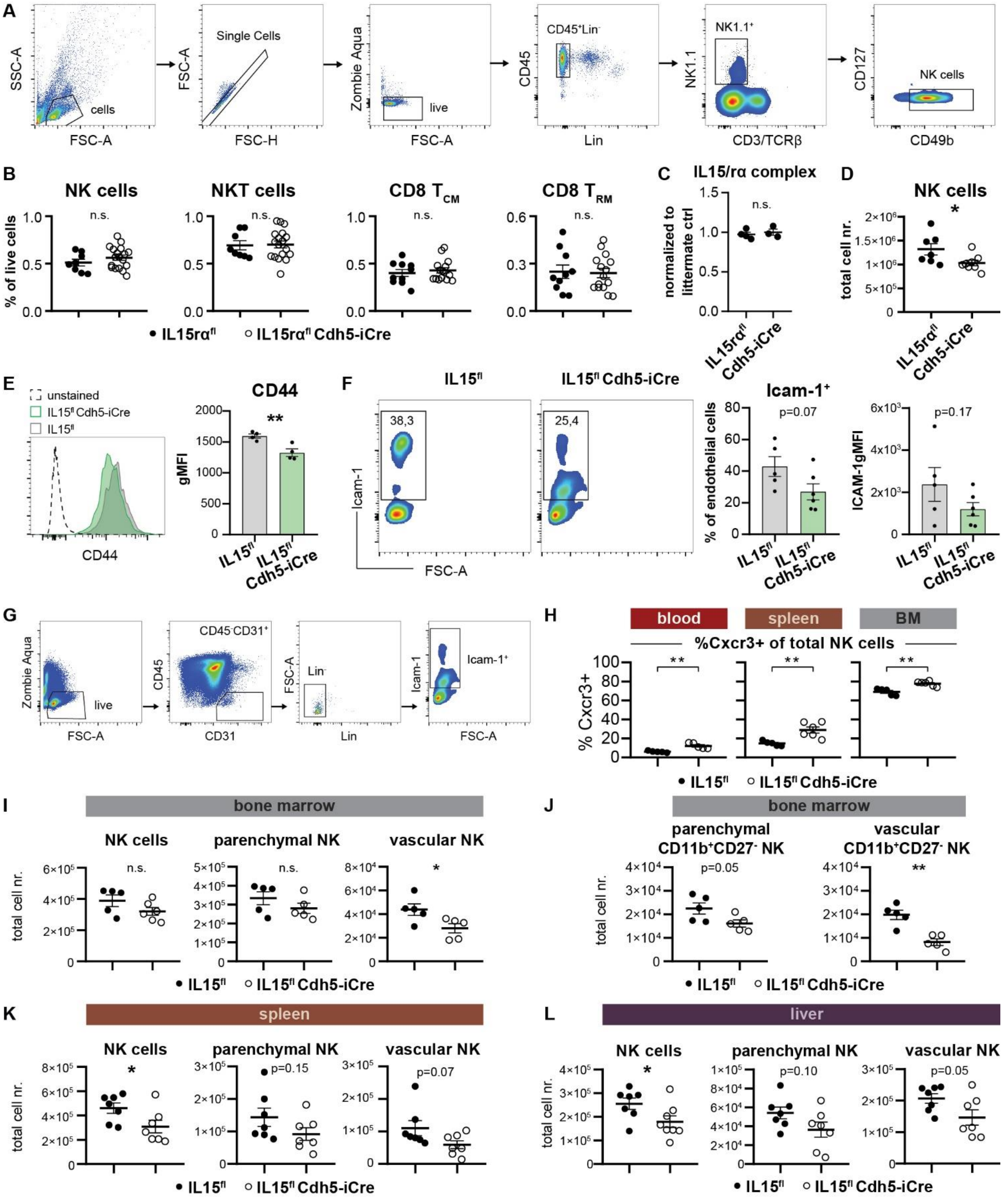

Supplementary Figure 5. Abundance of IL-15-dependent immune cell types in different organs of IL-15Rα^{flox} and IL-15^{flox} Cdh5-iCre mice, related to Figure 5.

(A) Flow cytometric gating strategy for (CD3$^-$NK1.1$^+$CD49b$^+$CD127$^-$) NK cells. **(B)** Relative quantification of NK cells (CD3$^-$NK1.1$^+$CD49b$^+$CD127$^-$), NKT cells (CD3$^+$NK1.1$^+$), central memory

$CD8^+$ T cells (CD8 T_{CM}) and tissue resident $CD8^+$ T cells (CD8 T_{RM}) in the bone marrow of tamoxifen-induced IL-15R$\alpha^{flox/flox}$ Cdh5-Cre/ERT2 (Cdh5-iCre) mice compared to IL-15R$\alpha^{flox/flox}$ littermates (IL-15Rα^{fl}). **(C)** ELISA comparing IL15/IL15RA complex (quantified as pg/mg total protein and normalized to littermate controls) in the bone marrow of IL-15R$\alpha^{flox/flox}$ Cdh5-Cre/ERT2 (Cdh5-iCre) mice and IL-15R$\alpha^{flox/flox}$ littermates (IL-15Rα^{fl}). **(D)** Absolute quantification of NK cells ($CD3^-$ $NK1.1^+CD49b^+CD127^-$) in the spleen, related to Figure 5A. **(E)** Mean fluorescence intensity of CD44 on blood NK cells (pre-gated on $CD3^-NK1.1^+$) from IL15$^{flox/flox}$ Cdh5-Cre/ERT2 (Cdh5-iCre) mice and IL15$^{flox/flox}$ (IL15^{fl}) littermates. **(F)** Frequency of ICAM-1^+ cells among endothelial cells (pre-gated on $CD45^-Ter119^-CD31^+$) and endothelial ICAM-1 MFI in the spleen of IL15$^{flox/flox}$ Cdh5-Cre/ERT2 (Cdh5-iCre) mice and IL15$^{flox/flox}$ (IL15^{fl}) littermates. **(G)** Gating strategy of Icam-1 expression in endothelial cells from digested spleens. The lineage (Lin) cocktail contains Ter119 and CD71 to exclude cells of the erythroid lineage. (**H**) Percentage of cells expressing CXCR3 among total NK1.1^+ NK cells in the blood, spleen and bone marrow of IL15$^{flox/flox}$ compared to IL-15$^{flox/flox}$ Cdh5-iCre mice. (**I**) Absolute quantification of parenchymal (CD45-PE^-) and vascular (CD45-PE^+) NK cells in the bone marrow of IL-15$^{flox/flox}$ and IL-15$^{flox/flox}$ Cdh5-iCre mice. (**J**) Cell numbers of parenchymal (CD45-PE^-) and vascular (CD45-PE^+) mature $CD11b^+CD27^-$ NK cells in the bone marrow of IL 15$^{flox/flox}$ Cdh5-iCre mice compared to IL 15$^{flox/flox}$ littermates. (**K**) Cell numbers of splenic NK cells in IL-15$^{flox/flox}$ versus IL-15$^{flox/flox}$ Cdh5-iCre mice, subdivided into parenchymal (CD45-PE^-) and vascular NK cells (CD45-PE^+). (**L**) Cell numbers of liver NK cells in IL-15$^{flox/flox}$ and IL-15$^{flox/flox}$ Cdh5-iCre mice, subdivided into parenchymal (CD45-PE^-) and vascular NK cells (CD45-PE^+). One animal was excluded from the vascular versus parenchymal quantification due to loss during the CD45-PE labeling procedure. Asterisks above the dot plots indicate P values from unpaired two-tailed Student's t tests. *$P < 0.05$, **$P < 0.01$, n.s. $P > 0.05$.

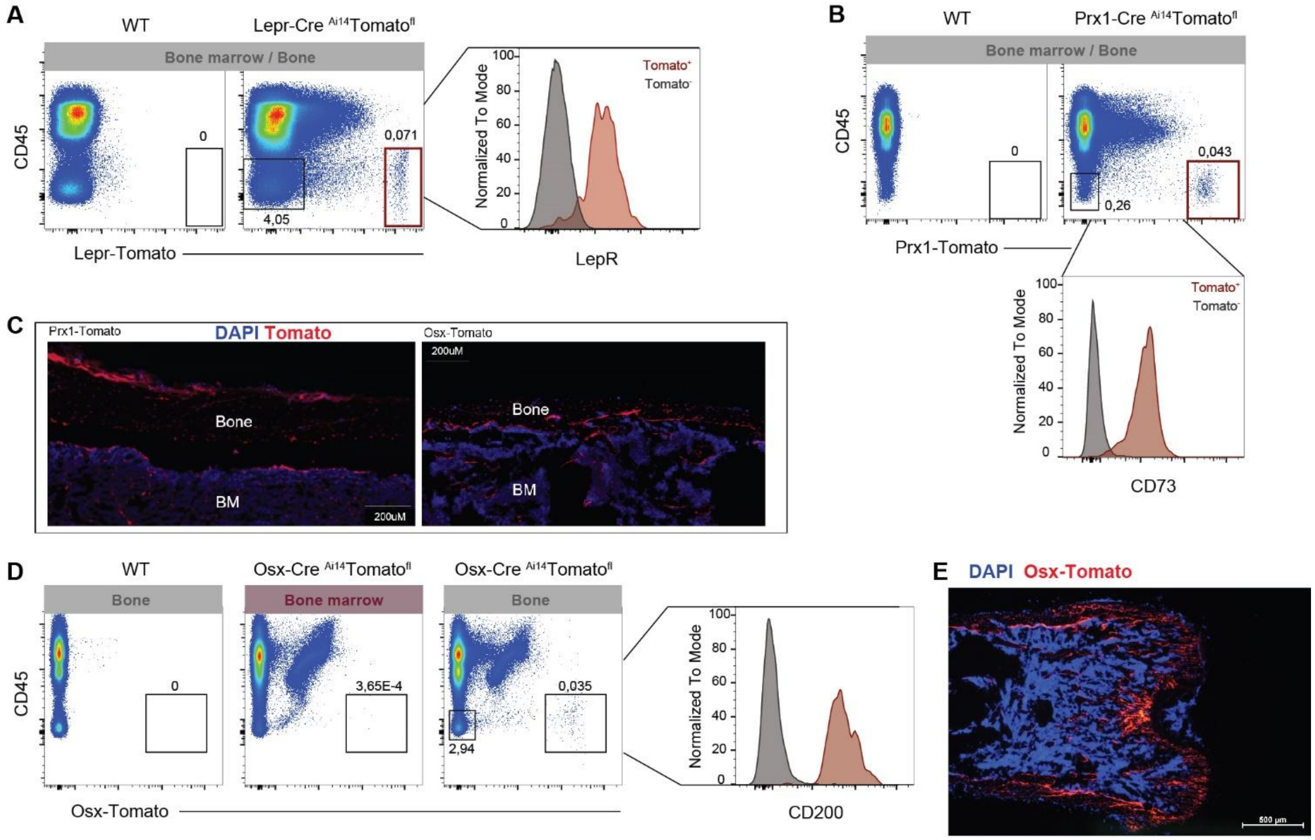

Supplementary Figure 6. Cre line reporter verification.

(A) Representative flow cytometry plots showing Tomato expression in stromal cells from WT and Lepr-Cre ^{Ai14}Tomato^{fl} mice (left) and Lepr protein expression in the Tomato^{+} and Tomato^{-} stromal fractions (right). **(B)** Flow cytometry plots showing Tomato expression in stromal cells from WT and Prx1-Cre ^{Ai14}Tomato^{fl} mice (top) and stromal CD73 marker expression in the Tomato^{+} and Tomato^{-} stromal fractions. **(C)** Immunofluorescence pictures showing DAPI and dsTomato reporter protein in femurs of Prx1-Cre ^{Ai14}Tomato^{fl} and Osx-Cre ^{Ai14}Tomato^{fl} mice with view of the bone marrow and cortical bone/periosteum. **(D)** Flow cytometry plots showing Tomato expression in stromal cells from bone or bone marrow of WT and Prx1-Cre ^{Ai14}Tomato^{fl} mice (left) and expression of the osteo-marker CD200 in the Tomato^{+} and Tomato^{-} stromal fractions from digested bone (right). **(E)** Immunofluorescence picture showing DAPI and dsTomato reporter protein in the femur of an Osx-Cre ^{Ai14}Tomato^{fl} mouse with Tomato being most dominantly expressed in cortical and trabecular bone and chondrocytes.

1.2 Supplementary Tables

Supplementary Table 1 - Primers

Primer name	type	Forward	Reverse
bAct	qPCR	GCTCTTTTCCAGCCTTCCTT	CTTCTGCATCCTGTCAGCAA
Cdh5-Cre/ERT2	genotyping	TCCTGATGGTGCCTATCCTC	CCTGTTTTGCACGTTCACCG
Cxcl12^{DsRed} KI	genotyping	ACGGCACCTTCATCTACCAC	ACCACGGTGTAGTCCTCGTT
Cxcl12^{DsRed} WT	genotyping	CTGGTTTTCGCCTCTAAAGC	CAGAGCTGCGAGCCTTTC
Il15	qPCR	GTGACTTTCATCCCAGTTGC	GCAAGGTAGAGCACGTTTC
Il15 flox	genotyping	AGAGCCTTGCCACTAGCTACA	AGAAGGCTTTGCAATGTTCA
Il15 flox deleted	genotyping	AGGATGCAGGAGATGTTTGG	CCTCTGCACCTTGACTGGTT
Il15^{GFP} KI	genotyping	ACTTCAAGATCCGCCACAAC	CACACACAGCCAAACACACA
Il15^{GFP} WT	genotyping	GCCACTTGGATCACATGAAA	CACACACAGCCAAACACACA
Il15ra	qPCR	GACACCAAAGGTGACCTCACAG	CTGTCTCTGTGGTCATTGCGGT
Il15ra deleted	genotyping	GGCAGTCAGCAAAGAAAGTTGG	CGATAAAGGTGGGCATCTACAGC
Il15ra flox	genotyping	GGCAGTCAGCAAAGAAAGTTGG	TTCCATCCATTCAGTGCGGC
IL-7 Cre	genotyping	CCGGGCTGCCACGACCAA	GGCGCGGCAACACCATTTTT
Lepr-Cre	genotyping	GCTGGAAGATGGCGATTAGC	TCTTCTTTCCAGAGTTCAGATGT
Lepr-WT	genotyping	CCCAATTTCAAACCTGTTCC	TCTTCTTTCCAGAGTTCAGATGT
Osx-Cre	genotyping	GAGAATAGGAACTTCGGAATAGTAAC	CCCTGGAAGTGACTAGCATTG
Osx-WT	genotyping	AGAGAGCTCCCCTCAATTATGT	AGCCACTTCTAGCACAAAGAACT
Prx1-control	genotyping	CTAGGCCACAGAATTGAAAGATCT	GTAGGTGGAAATTCTAGCATCATCC
Prx1-Cre	genotyping	GCGGTCTGGCAGTAAAAACTATC	GTGAAACAGCATTGCTGTCACTT
Rosa26 WT	genotyping	CTGGCTTCTGAGGACCG	CAGGACAACGCCCACACA
YFP mut	genotyping	AAGACCGCGAAGAGTTTGTC	AAAGTCGCTCTGAGTTGTTAT
YFP WT	genotyping	GGAGCGGGAGAAATGGATATG	AAAGTCGCTCTGAGTTGTTAT

Supplementary Table 2 – Details on the number, age and sex of mice used for experiments

**Figure 2B**					
**CD8 TCM**	n	females	males	age range	indep. experiments
WT	15	8	7	8-12wks	7
Cre	15	7	8	8-12wks	
**CD8 TEM**					
WT	13	7	6	8-10wks	6
Cre	13	6	7	8-10wks	
**CD8 TRM**					
WT	10	5	5	8-10wks	5
Cre	10	5	5	8-10wks	

**Figure 2G**					
**CD8 TCM**	n	females	males	age range	indep. experiments
WT	9	4	5	8-16wks	4
Cre	10	5	5	8-16wks	
**CD8 TEM**					
WT	9	4	5	8-16wks	4
Cre	10	5	5	8-16wks	
**CD8 TRM**					
WT	9	4	5	8-16wks	4
Cre	10	5	5	8-16wks	

**Figure 3B**					
**CD8 TCM**	n	females	males	age range	indep. experiments
WT	10	5	5	8-10wks	
Cre	9	4	5	8-10wks	8
KO	10	5	5	8-14wks	
**CD8 TEM**					
WT	10	5	5	8-10wks	
Cre	9	4	5	8-10wks	8
KO	10	5	5	8-14wks	
**CD8 TRM**					
WT	10	5	5	8-10wks	
Cre	9	4	5	8-10wks	8
KO	10	5	5	8-14wks	

**Figure 3C**					
**CD8 TCM**	n	females	males	age range	indep. experiments
WT	9	4	5	8-15wks	
Cre	7	3	4	8-15wks	4
KO	4	2	2	12-16wks	

**Figure 4A**					
**NK cells**	n	females	males	age range	indep. experiments
WT	9	5	4	8-20wks	6
Cre	17	8	9	8-20wks	
**NKT cells**					
WT	9	5	4	8-20wks	6
Cre	17	8	9	8-20wks	

**Figure 4B**					
**CD8 TCM**	n	females	males	age range	indep. experiments
WT	6	3	3	8-20wks	4
Cre	13	7	6	8-20wks	
**CD8 TRM**					
WT	6	3	3	8-20wks	4
Cre	13	7	6	8-20wks	

**Figure 4C**					
**CD8 TCM**	n	females	males	age range	indep. experiments
WT	5	3	2	8-20wks	3
Cre	10	5	5	8-20wks	
**NK cells**					
WT	8	4	4	8-20wks	3
Cre	9	4	5	8-20wks	

**Figure 4E**					
**$CD11b^{+}CD27^{-}$ NK cells**	n	females	males	age range	indep. experiments
WT	4	2	2	18-25wks	2
Cre	4	2	2	18-25wks	
**$KLRG1^{+}$ NK cells**					
WT	4	2	2	18-25wks	2
Cre	4	2	2	18-25wks	

**Figure 5A**					
**NK cells**	n	females	males	age range	indep. experiments
WT	10	2	8	10-20wks	3
Cre	12	2	10	10-20wks	

**Figure 5B**					
**CD8 TCM**	n	females	males	age range	indep. experiments
WT	10	2	8	10-20wks	3
Cre	12	2	10	10-20wks	

Figure 5C					
**NK cells**	n	females	males	age range	indep. experiments
WT	7	2	5	10-20wks	3
Cre	8	2	6	10-20wks	
**CD8 TCM**					
WT	7	2	5	10-20wks	3
Cre	8	2	6	10-20wks	

Figure 5D					
**NK cells**	n	females	males	age range	indep. experiments
WT	4	2	2	12-14wks	2
Cre	9	4	5	12-14wks	
**CD8 TCM**					
WT	4	2	2	12-14wks	2
Cre	9	4	5	12-14wks	

Figure 5E					
**NK cells**	n	females	males	age range	indep. experiments
WT	7	6	1	20-26wks	2
Cre	7	3	5	20-26wks	

Figure 5F-G					
**NK cells, parenchymal NK, vascular NK**					
	n	females	males	age range	indep. experiments
WT	5	4	1	20-26wks	2
Cre	5	2	4	20-26wks	

Figure 5H-I					
**NK cells, parenchymal NK, vascular NK**					
	n	females	males	age range	indep. experiments
WT	7	6	1	20-26wks	2
Cre	7	3	5	20-26wks	
